# Ecophysiological and biochemical responses to cold and heat waves of native *Spartina maritima*, alien *S. densiflora* and their reciprocal hybrids

**DOI:** 10.1007/s00425-025-04675-4

**Published:** 2025-04-01

**Authors:** Rosario Álvarez, Salvador A. Fernandez-Gonzalez, Adrián Perera-Bonaño, Alfonso De Cires, Jesús M. Castillo, Blanca Gallego-Tévar

**Affiliations:** Departamento de Biología Vegetal y Ecología, Universidad de Sevilla, Ap 1095, 41080 Seville, Spain

**Keywords:** Anthocyanins, Climate change, Cordgrass, Hybridization, Mediterranean climate, Thermal stress

## Abstract

**Main conclusion:**

*Spartina* hybrids outperform parental species, showing transgressive acclimation to extreme climates. Native *S. maritima* demonstrates high seasonal adaptability and invasive *S. densiflora* low physiological impact, suggesting resilience under climate change.

**Abstract:**

Extreme climatic events, such as cold and heat waves, are becoming more frequent, intense, and prolonged due to climate change. Simultaneously, invasive alien plant species are altering the composition of plant communities. Both climate change and the introduction of alien species pose significant threats to biodiversity. We studied the responses of 25 biochemical and physiological functional traits for native *Spartina maritima*, alien invasive *S. densiflora* and their reciprocal hybrids to changing environmental conditions during a cold snap in winter and a heat wave in summer in Guadiana Marshes (Southwest Iberian Peninsula). These four closely related taxa responded differently to seasonal environmental fluctuations. Both hybrid taxa, particularly *S. maritima* × *densiflora*, exhibited transgressive responses, allowing them to display a wider range of acclimation responses to air temperature compared to their parental species. Native *S. maritima* also demonstrated a relatively high acclimation capacity to seasonal meteorological changes. In contrast, alien *S. densiflora* presented few acclimation responses to seasonal environmental changes, responding primarily to sediment salinity rather than to air temperature. Even so, all four studied *Spartina* taxa appear to be well-adapted to the occurrence of cold and heat waves in the Gulf of Cadiz. These findings underscore the complexity of plant acclimation strategies in response to extreme climatic events and highlight the potential for hybrid taxa to face the future dynamics of salt marshes under climate change.

**Supplementary Information:**

The online version contains supplementary material available at 10.1007/s00425-025-04675-4.

## Introduction

Extreme climatic events, such as cold and heat waves, are increasing in frequency, intensity and duration in the present scenario of climate change (IPCC 2022). These climatic changes are especially relevant for the Mediterranean Basin which has been identified as a climate change hotspot (Noto et al. 2023). Cold and heat waves expose plants to high levels of thermal stress that can diminish photosynthesis, a highly sensitive process to temperature changes (Chovancek et al. 2019; Grossman 2023). Thermal stress may reduce the efficiency of photosynthetic electron transport in both photosystems and alter pigment accumulation and chlorophyll (Chl) fluorescence quenchings (Teskey et al. 2015; Nievola et al. 2017). Diminished photosynthesis as a result of thermal stress can reduce biomass production and the capacity to face environmental stresses that, in turn, may cause broad biogeographic shifts and regional changes in distribution (Aagesen et al. 2016; Liancourt et al. 2020).

Together with climate change, invasive alien plant species are changing species composition of plant communities and constitute also a severe threat to biodiversity (Fried et al. 2014; van Kleunen et al. 2018). Some alien plant species can suffer from non-reversible photodamage and reduced photosynthesis due to thermal stress (Zhu et al. 2019). In contrast, many alien species may exhibit enhanced responses, at biochemical and photochemical levels, to thermal stress than their native congeners (Duarte et al. 2016; Kunert et al. 2022). The physiological challenges posed by climate change for native and alien plant species can be faced by phenotypic plasticity and local adaptation. In general, invasive species present higher phenotypic plasticity in their photosynthesis performance than non-invasive species (Davidson et al. 2011). Moreover, some alien plant species have adapted a wide range of biochemical and physiological traits to the climate in their introduced range, facilitating extensive invasions (Fenollosa and Munne-Bosch 2019). In this context, rapid global warming has enabled alien species to expand into regions in which they previously could not survive and reproduce (Walther et al. 2009; Kathiresan and Gualbert 2016). Liu et al. (2017) found that elevated temperature and CO_2_ enrichment favoured invasive alien plants more strongly than native plants. Nevertheless, in general, both invasive alien plants and their native counterparts usually undergo similar adaptive evolution responses of physiological performance to global warming (Gianoli et al. 2021).

Alien plant species can hybridize with native species and the upcoming hybrids can show increased fitness in responses to different environmental stresses due to biochemical and physiological transgressive traits (Gallego-Tévar et al. 2019a, 2020a, b; Zhang et al. 2020). In this context, Sun et al. (2015) found that hybridization slightly catalyzed the tolerance of a hybrid between native and alien *Sphagneticola* species to low temperature and weak light conditions, recorded as changes in photosynthetic and antioxidant functional traits. Maternal effects also play a relevant role in the functional trait expression of interspecific hybrids facing environmental stress (Filipe and Montesinos 2016), including the expression of photosynthetic traits (Ji and Jiao 1999). Moreover, the formation of these hybrids can be modulated by increasing temperatures in the present scenario of global warming (Gallego-Tévar et al. 2019b). Therefore, hybridizations between native and alien species are of great concern to evolutionary biologists and ecologists interested in quick evolutionary processes.

Understanding the biochemical and physiological responses of native and alien plant congener species and their hybrids to seasonal meteorological changes and extreme climatic events is crucial for predicting how these taxa might cope with future environments in the context of climate change. To our knowledge, none study has analyzed the biochemical and physiological responses of native and invasive congeners and their hybrids to cold and heat waves under field conditions. Field studies are especially important since they capture the intricate interplay of environmental factors influencing plant performance and resilience. For example, plant growth-promoting bacteria found in natural conditions can improve the tolerance of the photosynthetic apparatus to thermal stress (Duarte et al. 2023).

Our study system was the native cordgrass *Spartina maritima* (Curtis) Fernald (2n = 60), the alien invasive *Spartina densiflora* Brongn. (2n = 70) and their reciprocal hybrids growing in Guadiana Marshes (Gulf of Cadiz, southwest Iberian Peninsula). *Spartina maritima* is a foundation species and a marsh-building halophyte that dominates Iberian and other European and South African low salt marsh zones (Castellanos et al. 1994). Alien *Spartina densiflora* is a neophyte with high phenotypic plasticity (Castillo et al. 2018) that is invading contrasting habitats along the tidal gradient in the Gulf of Cadiz (Nieva et al. 2001). The hybrids *S. maritima* × *densiflora* (2n = c. 95) and *S. densiflora* × *maritima* (2n = 65) are sterile hybrids that show some transgressive traits in the field (eg. taller shoots and higher growth rates than the parental species) (Castillo et al. 2010) and in response to salt stress under controlled conditions (Gallego-Tévar et al. 2018a, 2019a). We studied the responses of 23 biochemical and physiological functional traits of our four focal *Spartina* taxa to changing environmental conditions during a cold snap in winter and a heat wave in summer. We hypothesized that both parental species would show high tolerance to changing environmental conditions through acclimation of different functional traits and that both hybrids would present transgressive traits enabling them to show higher acclimation capacity than parental species.

## Materials and methods

### Study area

Our study was carried out in the San Bruno Marshes (37º10ʹ−37º16ʹN, 7º28ʹ−7º16ʹW), located in the southwest of the Iberian Peninsula, in the Guadiana River Marshes (Fig. S1). The Guadiana Estuary is a mesotidal open estuary with semidiurnal tides, a mean range of 2.10 m and a mean spring tidal range of 2.97 m (Castellanos et al. 1994). The study area presents a gentle slope along a wide tidal gradient, showing a clear plant zonation pattern with low marshes dominated by *Spartina maritima* and *Sarcocornia perennis* (Mill.) A.J. Scott, middle marshes by *Halimione portulacoides* (L.) Aellen, *Sarcocornia fruticosa* (L.) A.J. Scott and exotic *S. densiflora*, and high marshes by *Arthrocnemum macrostachyum* (Moric.) C. Koch and *Limoniastrum monopetalum* (L.) Boiss. (Gallego-Tévar et al. 2018b). The study area has a Mediterranean climate with Atlantic influence with mild and relatively wet winters (mean air temperature is ca. + 11 ºC in January; average annual precipitation is c. 506 mm), and hot and dry summers (mean air temperature is + 25 ºC in August with almost no rainfall). The studied Mediterranean salt marshes are exposed to broad daily and seasonal changes in temperature (> 30 °C) and very high temperatures during summertime (> + 40 °C) (Boughalleb et al. 2022). Sediment salinity levels peak near seawater concentration during summer droughts (Contreras-Cruzado et al. 2017). Additionally, the studied Mediterranean salt marshes are exposed to sea level rise (+ 4.02 mm yr^−1^ and accelerating) and reduced rainfalls due to climate change (Kovats et al. 2014; NOAA 2018).

### Environmental matrix

Meteorological data (minimum, average and maximum air temperature, ºC) for the sampling days and previous 9 days in winter and summer were collected from Ayamonte meteorological station, located 2 km away from the studied marshes (code 4549Y; AEMET 2024). The sedimentary environment was characterized at the same time that leaf samples were collected for laboratory analyses. We recorded sediment redox potential (Eh; mV), pH, electrical conductivity (EC; mS cm^−1^) and water content (WC; %) in the root zone, between 0 and 10 cm deep, of every marked *Spartina* clump (*n* = 10 samples per taxon). Eh was recorded in situ using an electrode system (Crison Instruments pH/mV p-506, Hach Lange Spain, S.L.U., Barcelona, Spain) (Castillo et al. 2000). Sediments (250 ml) were sampled in sealed plastic containers and transported to the laboratory. Sediment pore-water pH and EC were recorded in the unfiltered supernatant after adding distilled water to the sediment samples (1:1, v/v) using a pHmeter (pH/redox Crison PH25 with the probe Crison 5052) and a conductivity meter (Crison CM35), respectively, in the laboratory. Sediment WC was recorded by weighting ca. 100 g of sediments before and after drying to constant weight at + 80 ºC in a forced-air stove (Curado et al. 2014).

### Plant material

Field measurements and sample collections took place during low tidal level around solar noon on sunny days and low tide conditions in January 2021 during a cold snap associated with Storm Filomena and in July 2021 during a heat wave (defined by the Spanish Meteorological State Agency (AEMET; acronym in Spanish) as ≥ 3 days with temperatures above the 95th percentile for July–August 1971–2000). All measurements were carried out on the first totally expanded adult leaf from adult shoots of ten randomly selected clumps of each taxon: native *Spartina maritima*, alien invasive *S. densiflora* and their reciprocal hybrids *S. maritima* × *densiflora* and *S. densiflora* × *maritima*. Each taxon was sampled along the tidal gradient there where it was more abundant. *Spartina maritima* and *S. maritima* × *densiflora* were sampled in low marshes, and *S. densiflora* and *S. densiflora* × *maritima* in middle marshes. Sampled clumps were separated at least 2 m from each other to ensure they were different individual plants that were permanently marked.

### Crude extract for laboratory analyses

First, totally developed adult leaves (flag leaves) were collected from 5 randomly selected plants from the 10 marked plant of each taxa (*n* = 5 samples per taxon), immediately stored in zip-lock plastic bags with silica gel in the field and stored at − 20 ºC once in the laboratory. To obtain the crude extract, 0.2 g of leaves were weighed and processed in mortar during 10–15 min with 10 ml of pure methanol (using sand to help extraction). To eliminate any solid residue, samples were centrifuged at 9981 g, 15 min at 10 ºC. Supernatant was used as a crude extract for the determinations of pigments, total antioxidant capacity, malondialdehyde (MDA) and polyphenol concentrations.

### Pigments determination

The concentrations of photosynthetic pigments and anthocyanins are modified in response to thermal stress (Vetoshkina et al. 2023). To assess chlorophylls (Chl) and carotenoids (Car) concentrations from the crude extract (previously diluted 10 times in pure methanol), absorbance (Abs) at 665.2 nm, 652.4 nm and 470 nm was measured. Chl *a*, *b* and carotenoids were calculated using the following equations (Lichtenthaler and Buschmann 2001):

$${\text{Chl }}a{ }\left( {{\mu g}/{\text{ml}}} \right) = 16.72{\text{ Abs}}_{665.2} - 9.16{\text{ Abs}}_{652.4}$$

$${\text{Chl }}b{ }\left( {{\mu g}/{\text{ml}}} \right) = 34.09{\text{ Abs}}_{652.4} - 15.28{\text{ Abs}}_{665.2}$$

$${\text{Car }}\left( {{\mu g}/{\text{ml}}} \right) = \left( {1000{\text{ Abs}}_{470} - 1.63{\text{ Chl }}a - 104.96{\text{ Chl }}b} \right)/221$$

Anthocyanin concentration was measured diluting the crude extract with HCl until the final concentration reached 1% (v/v). Samples were kept at 4 ºC in darkness. After 24 h, absorbance was measured at 530 nm and 653 nm. Anthocyanin concentration was determined according to Mancinelli et al. (1974). These and all other UV/visible spectrophotometric assays described below were carried out using a UV-3100PC spectrophotometer (VWR).

### Polyphenols

Many plant species synthesize and accumulate phenolic compounds as a defense mechanism against environmental stressors (Yang et al. 2018). Phenolic compounds were assayed using the Folin-Ciocalteu reagent following Singleton and Rossi (1965). To assess polyphenol concentrations, 0.5 ml of Folin–Ciocalteau and 4.5 ml of distilled water were added to 1 ml of crude extract (previously diluted in distilled water in a 7:3 ratio). Samples were put in darkness at room temperature. After 8 min, 4 ml of 7.5% (w/v) sodium carbonate (Na_2_CO_3_) was added to each sample. After 1-h incubation under the same conditions absorbance at 765 nm was measured. Polyphenol concentrations were expressed in mg of gallic acid (GA) g DW^−1^ (Slama et al. 2017). GA was used as a standard compound. GA stock solution (20 mg/100 ml methanol) was prepared and various dilutions were obtained for the standard calibration curve.

### Total antioxidant capacity

Reduction of Mo (VI) to Mo (V) at acid pH produces a turquoise compound that can be measured to assess total antioxidant capacity. To record total antioxidant capacity 1 ml of reagent (0.6 M sulfuric acid, 28 mM sodium phosphate, 4 mM ammonium molybdate) was added to 0.1 ml of crude extract (previously diluted in distilled water in a 7:3 ratio). Samples were kept at 95 ºC for 90 min. After cooling for 20 min at room temperature, absorbance at 695 nm was measured against a blank. Total antioxidant capacity was expressed in mg GA/g DW^−1^ (Prieto et al. 1999; Slama et al. 2017).

### Malondialdehyde determination

MDA, a biomarker of lipid peroxidation, indicates oxidative damage resulting from abiotic stresses (Kim et al. 2017). To assess MDA concentration as a lipid peroxidation index, we followed the protocol described by Hodges et al. (1999) with some modifications (Taulavuori et al. 2001). A total of 2.5 ml of crude extract was divided into two test tubes, each containing 1.25 ml. We added 1.5 ml of 20% (w/v) trichloroacetic acid (TCA) to the first tube, while the second tube received 1.5 ml of a solution containing 0.5% (w/v) thiobarbituric acid (TBA) in 20% (w/v) TCA. Then, 0.25 ml of pure methanol was added to each tube. After a 15-min incubation at 95 °C, samples were placed on ice to terminate the reaction. After centrifuging samples at 9981 g for 10 min, the supernatant's absorbance was measured at 440 nm, 532 nm, and 600 nm. MDA concentration was calculated using the following equations:

$${\text{A }} = {\text{ Abs 532 }}{-}{\text{ Abs 6}}00 \, {-} \, \left( {{\text{Abs }}532_{Only TCA} {-}{\text{ Abs }}600_{Only TCA} } \right)$$

$${\text{B }} = \, \left( {{\text{Abs 44}}0 \, {-}{\text{ Abs 6}}00} \right) \, * \, 0.0{571}$$

$${\text{MDA}}\left( {\frac{nmol}{{mL}}} \right) = \left( {\frac{A - B}{{157000}}} \right) \, *{ 10}^{6}$$

### Apical leaf growth

Apical leaf growth serves as an effective measure for quantifying stress responses in *Spartina* taxa in field conditions (Castillo et al. 2014). Apical leaf growth was measured marking 3–5 leaves per clump with permanent sealant at their base, and measuring the distance from the sealant to the leaf base 2 days later for the ten marked clumps per taxon (*n* = 10 samples per taxon).

### Maximal rate of photosynthetic oxygen evolution

Maximal rate of photosynthetic oxygen evolution (*V*_max_) has been previously recorded to quantify foliar thermal stress in halophytes (Figueroa-Luque et al. 2024). We measured *V*_max_ to obtain information on the state of the photosynthetic apparatus under forced conditions of CO_2_ saturation and high radiation level. Leaves were sampled from the ten marked clumps per taxon in the evening when the tide was raising and just before the study plants were being inundated (*n* = 10 samples per taxon). Then leaves were stored in a water vapor-saturated atmosphere at 25 °C at darkness and 4 ºC until measurements were carried out early in the morning. *V*_max_ measurements were carried out using an oxygen electrode type Clark (Hansatech LD2, Pentney, UK) at 25 °C and photosynthetic photon flux density (PPFD) 1400 µmol m^– 2^ s^−1^ in a CO_2_-saturated atmosphere obtained with a 1 M carbonate/bicarbonate buffer (pH = 9). *V*_max_ was recorded as a release of O_2_ per unit of time and g FW (µmolO_2_ s^−1^ gFW^−1^) (*n* = 10 measurements per taxon) (Farquhar et al. 2001; Popova et al. 2019).

### Chlorophyll fluorescence

Chlorophyll *a* fluorescence is a useful tool to assess the effects of thermal stress on the photosynthetic apparatus (Kunert 2024). Light and dark-adapted Chl fluorescence were measured in one leaf per each of the 10 marked clumps per taxon (*n* = 10 samples per taxon) at sunrise (PPFD ca. 200 µmol m^−2^ s^−1^) and at noon (PPFD of 1100–2100 µmol m^−2^ s^−1^) with a portable modulated fluorimeter (FMS-2, Hansatech Instruments) using leaf clips for dark adaptation for 30 min. Noon measurements inform about the levels of total (dynamic and permanent) photoinhibition, while sunrise measurements record the permanent photoinhibition levels that has not been able to recover during the night (Fernández-Baco et al. 1998). Chl fluorescence parameters were measured according to Maxwell and Johnson (2000). Initial fluorescence (F_0_) in the dark-adapted state was measured using a PPFD < 0.05 µmol m^–2^ s^−1^ for 1.8 µs, too small to induce significant physiological changes in the plant. Maximal fluorescence (F_m_) was recorded after a saturating light pulse of 15.000 µmol photons m^−2^ s^−1^. Variable fluorescence (F_v_ = F_m_ − F_0_) and maximum quantum efficiency of photosystem II (PSII) photochemistry (F_v_/F_m_) were calculated to quantify photoinhibition. Using the same leaf section, light-adapted parameters were measured before assessing dark-adapted Chl fluorescence in leaves acclimated to full solar radiation. Steady-state fluorescence yield (F_s_) was recorded after adapting plants to ambient light conditions (with full sunlight of 1150 µmol photons m^−2^ s^−1^). A saturating actinic light pulse of 15,000 µmol photons m^−2^ s^−1^ for 0.7 s was then used to produce the maximum fluorescence yield (F_m_´) by temporarily inhibiting PSII photochemistry. Effective quantum efficiency of PSII (Φ_PSII_ = (F_m_´—F_s_)/F_m_´) was calculated. Non-photochemical quenching (NPQ) was calculated from parameters obtained in both dark and light-adapted states.

### Data analyses

#### Inheritance mechanisms

To investigate the inheritance mechanisms underlying salt stress responses, the above-described plant traits were analyzed in the reciprocal hybrids of *S. maritima* and *S. densiflora*. The following inheritance mechanisms were identified, as described by Favre and Karrenberg (2011). (1) Dominant inheritance, when a hybrid exhibited a trait similar to one of its parents. This was denoted as “D-Sm” for *S. maritima* or “D-Sd” for *S. densiflora*. (2) Parental codominance when a hybrid's trait was not different from the two parents, represented as “D-Sm,Sd”. (3) Parental additivity, when a hybrid trait fell within the range of its parents but was significantly different from both. (4) Transgressive segregation, when hybrids exhibited traits that exceeded, by at least 5%, the phenotypic range of both parents. These inheritance mechanisms were quantified for each plant trait in both hybrid populations and the differences between species were based on the significant differences exerted for each variable (see next section).

### Statistical analyses

All statistical analyses were carried out using R software (R-core team 2024). We applied a significance level (α) of 0.05 for every analysis. Deviations of all data were calculated as the standard error of the mean (SE). Plant traits were classified into two functional groups: (1) biochemical stress responses (anthocyanins, Chl *a*, Chl *b*, carotenoids, MDA, polyphenols and antioxidant capacity), and (2) ecophysiological and growth responses (F_0_, F_m_, Fv, F_v_/F_m_, F_s_, F_m_´, Φ_PSII_ and NPQ at sunrise and noon, *V*_max_ and apical leaf growth). To protect analyses from type I error, the means of the dependent variables of each trait group were compared using multivariate analysis of variance (MANOVA) and Wilks’ Lambda to evaluate the significance of the factors taxon (*S. maritima*, *S. densiflora*, *S. maritima* × *densiflora* and *S. densiflora* × *S. maritima*) and season (winter and summer) (Scheiner 2001). Redundant, highly correlated variables (r > 0.90) were identified prior to MANOVA analysis and were omitted from the statistical models (F_m_ at sunrise and noon). Once multivariate significance was confirmed via MANOVA, the main univariate differences of plant traits were evaluated for each plant trait with General Lineal Models (GLMs) and Bonferroni-Dunn's test as a post hoc analysis. Environmental variables were also analyzed using GLMs with *taxa* and *season* as fixed factors and Bonferroni-Dunn's test as a post hoc analysis. The adequacy of model assumptions for the GLMs, including checking for uniformity and residual diagnostics, was assessed using the *DHARMa* package (Hartig 2022). Environmental variables were also compared between seasons and taxa using GLMs and Bonferroni-Dunn's test as a post hoc analysis.

We performed Canonical Correspondence Analyses (CCA), using the *vegan* package (Oksanen et al. 2024), to identify the environmental variables that most greatly influenced plant traits in the studied *Spartina* taxa. CCA was conducted using a full model to test the significance of the relationships between the environmental variables measured in winter and summer and the plant traits matrix for each taxon. Monte-Carlo permutation tests (999 permutations) were performed for assessing the significance of the canonical correlation coefficients. Average daily maximum and minimum air temperatures were removed during the analysis due to their multicollinearity with average daily mean air temperature.

## Results

### Environmental matrix

The study area presented marked seasonal air temperatures and rainfall was concentrated mainly from September to February during the sampling year (Fig. S2). Mean minimum daily air temperature was 21 ºC lower during the winter cold snap than in the summer heat wave. This seasonal difference was 10 ºC for average daily temperature and 19 ºC for maximum daily temperature. Mean sediment Eh was always higher than 95 mV for every taxon in both seasons, being higher in winter than in summer. Sediment WC did not present seasonal differences, and varied between 45 ± 1% for *S. densiflora* × *maritima* in winter and 72 ± 2% for *S. maritima* × *densiflora* in summer. Sediment EC was higher in summer than in winter, and varied between 8.7 ± 0.5 mS cm^−1^ for *S. densiflora* in winter and 16.7 ± 0.5 mS cm^−1^ for *S. densiflora* × *maritima* in summer. Mean sediment pH was higher in winter than in summer and ranged between 6.7 and 7.5 (Tables 1 and 2).

**Table 1:** F-statistic and *P-*values of G*eneral Linear Models* with taxon (T), season (S) (winter and summer) and their interaction (T x S) as fixed factors, for environmental variables measured in individuals of *Spartina maritima*, *Spartina densiflora* and their reciprocal hybrids in the Guadiana Marshes (Southwest Iberian Peninsula)

Environmental variables	Taxon (T)		Season (S)		T × S	
	F_3,72_	*P*	F_1,18–72_	*P*	F_3,72_	*P*
Minimum daily air temperature (ºC)	–	–	**294.63**	** < 0.0001**	–	–
Average daily air temperature (ºC)	–	–	**873.15**	** < 0.0001**	–	–
Maximum daily air temperature (ºC)	–	–	**536.83**	** < 0.0001**	–	–
Sediment redox potential (mV)	**3.888**	**0.012**	**4.657**	**0.034**	0.267	0.849
Sediment water content (%)	**37.139**	** < 0.0001**	1.105	0.297	**4.534**	**0.006**
Sediment electrical conductivity (mS cm^−1^)	**16.204**	** < 0.0001**	**11.921**	**0.001**	0.017	0.997
Sediment pH	**52.956**	** < 0.0001**	**50.579**	** < 0.0001**	2.403	0.075

Values for F-statistics with degrees of freedom (*n*) as subscripts are displayed. Significant differences are highlighted in bold

**Table 2:** Meteorological and sedimentary environmental factors for native *Spartina maritima*, introduced *S. densiflora* and their reciprocal hybrids in the Guadiana Marshes (Southwest Iberian Peninsula)

	Winter				Summer			
	*S. maritima*	*S. maritima* × *densiflora*	*S. densiflora*	*S. densiflora* × *maritima*	*S. maritima*	*S. maritima* × *densiflora*	*S. densiflora*	*S. densiflora* × *maritima*
Minimum daily air temperature (ºC)	4.0 ± 0.6^a^				25.2 ± 0.4^b^			
Average daily air temperature (ºC)	8.8 ± 0.4^a^				18.7 ± 0.6^b^			
Maximum daily air temperature (ºC)	13.3 ± 0.6^a^				31.7 ± 0.5^b^			
Sediment redox potential (mV)	167 ± 22	175 ± 24	189 ± 18	125 ± 11	124 ± 21	168 ± 28	149 ± 18	96 ± 15
Sediment water content (%)	70 ± 2	70 ± 2	71 ± 2	45 ± 1	67 ± 2	72 ± 2	68 ± 3	56 ± 2
Sediment pH	7.5 ± 0.0	7.4 ± 0.0	7.5 ± 0.0	6.9 ± 0.0	7.1 ± 0.1	7.3 ± 0.1	7.2 ± 0.1	6.7 ± 0.0
Sediment electrical conductivity (mS cm^−1^)	9.1 ± 0.7	9.0 ± 0.9	8.7 ± 0.5	14.5 ± 1.0	11.3 ± 1.1	11.4 ± 1.3	11.2 ± 1.2	16.7 ± 0.5

Values are mean ± SE (*n* = 10). Different letters indicate significant seasonal differences for a given taxon (General Lineal Models and Bonferroni-Dunn's test as a post hoc analysis; see Table 1)

### Plant responses

*Spartina maritima*, *S. densiflora* and both reciprocal hybrids exhibited seasonal variations in different biochemical, ecophysiological and growth traits (Tables 3 and 4).

**Table 3:** Wilks’ lambda, F-statistic, degrees of freedom and *P*-values from MANOVAs for the two trait response groups for the factors *season* (winter and summer), *taxon* (*S. maritima, S. densiflora, S. maritima* × *densiflora* and *S. densiflora* × *maritima*) and their interaction

	Factors	Wilks’ Lambda	F	df	*P*
Biochemistry	Season	0.165	18.832	7	< 0.001
	Taxon	0.023	0.984	21	< 0.001
	Season*Taxon	0.057	6.120	21	< 0.001
Ecophysiology and growth	Season	0.042	81.726	16	< 0.001
	Taxon	0.094	4.308	48	< 0.001
	Season*Taxon	0.233	2.240	48	< 0.001

The higher Wilks’ lambda is, the stronger the evidence that the independent variables (factors) have a statistically significant effect on the dependent variable

**Table 4:** F-statistic and *P-*values of G*eneral Linear Models* with taxon (T), season (S) (winter and summer) and their interaction (T x S) as fixed factors, for biochemical and ecophysiological and growth-related traits measured in individuals of *Spartina maritima*, *Spartina densiflora* and their reciprocal hybrids in the Guadiana Marshes (Southwest Iberian Peninsula)

	Plant traits	Taxon (T)		Season (S)		T × S	
		F_3,32_	*P*	F_1,32_	*P*	F_3,32_	*P*
Biochemistry	Anthocyanins (µg g DW^−1^)	**9.28**	** < 0.001**	**49.33**	** < 0.001**	2.05	0.126
	Chl *a* (µg g DW^−1^)	**12.45**	** < 0.001**	**12.00**	** < 0.01**	2.17	0.111
	Chl *b* (µg g DW^−1^)	**4.82**	** < 0.001**	2.29	0.140	0.68	0.572
	Carotenoids (µg g DW^−1^)	**8.17**	** < 0.001**	**17.69**	** < 0.001**	**10.90**	** < 0.001**
	MDA (nmol g DW^−1^)	2.263	0.100	**4.58**	** < 0.05**	**6.13**	** < 0.01**
	Polyphenols (mg g DW^−1^)	**66.12**	** < 0.001**	**15.81**	** < 0.001**	**6.08**	** < 0.01**
	Antioxidant capacity (mg g DW^−1^)	**17.75**	** < 0.001**	**71.53**	** < 0.001**	**20.62**	** < 0.001**
		F_3,72_	*P*	F_1,72_	*P*	F_3,72_	*P*
Ecophysiology and growth	Apical leaf growth (cm)	**21.37**	** < 0.001**	**35.68**	** < 0.001**	0.54	0.653
	*V*_max_ (μmol O_2_ g^−1^ FW^−1^)	**6.20**	** < 0.001**	**26.16**	** < 0.001**	0.61	0.608
	F_0_ sunrise	**2.77**	** < 0.05**	1.49	0.226	2.00	0.121
	F_v_ sunrise	**6.57**	** < 0.001**	**22.83**	** < 0.001**	**5.78**	** < 0.01**
	F_v_/F_m_ sunrise	**6.22**	** < 0.001**	**13.91**	** < 0.001**	1.29	0.284
	F_s_ sunrise	**2.90**	** < 0.05**	**9.28**	** < 0.01**	**4.31**	** < 0.01**
	F_m_’ sunrise	**3.09**	** < 0.05**	**103.30**	** < 0.001**	**6.89**	** < 0.001**
	ΦPSII sunrise	**6.36**	** < 0.01**	**726.75**	** < 0.001**	**3.39**	** < 0.05**
	NPQ sunrise	1.44	0.238	**25.19**	** < 0.001**	2.50	0.066
	F_0_ noon	0.85	0.470	**7.53**	** < 0.01**	0.89	0.451
	F_v_ noon	**4.32**	** < 0.01**	**21.16**	** < 0.001**	**4.64**	** < 0.01**
	F_v_/F_m_ noon	2.63	0.056	**4.12**	** < 0.05**	2.38	0.077
	F_s_ noon	2.21	0.094	1.63	0.205	**5.22**	** < 0.01**
	F_m_’ noon	2.07	0.112	0.55	0.459	**4.80**	** < 0.01**
	ΦPSII noon	1.01	0.392	**15.62**	** < 0.001**	1.47	0.229
	NPQ noon	1.13	0.341	3.32	0.0725	**3.80**	** < 0.05**

Significant differences are highlighted in bold

### Pigments

**Fig. 1:**
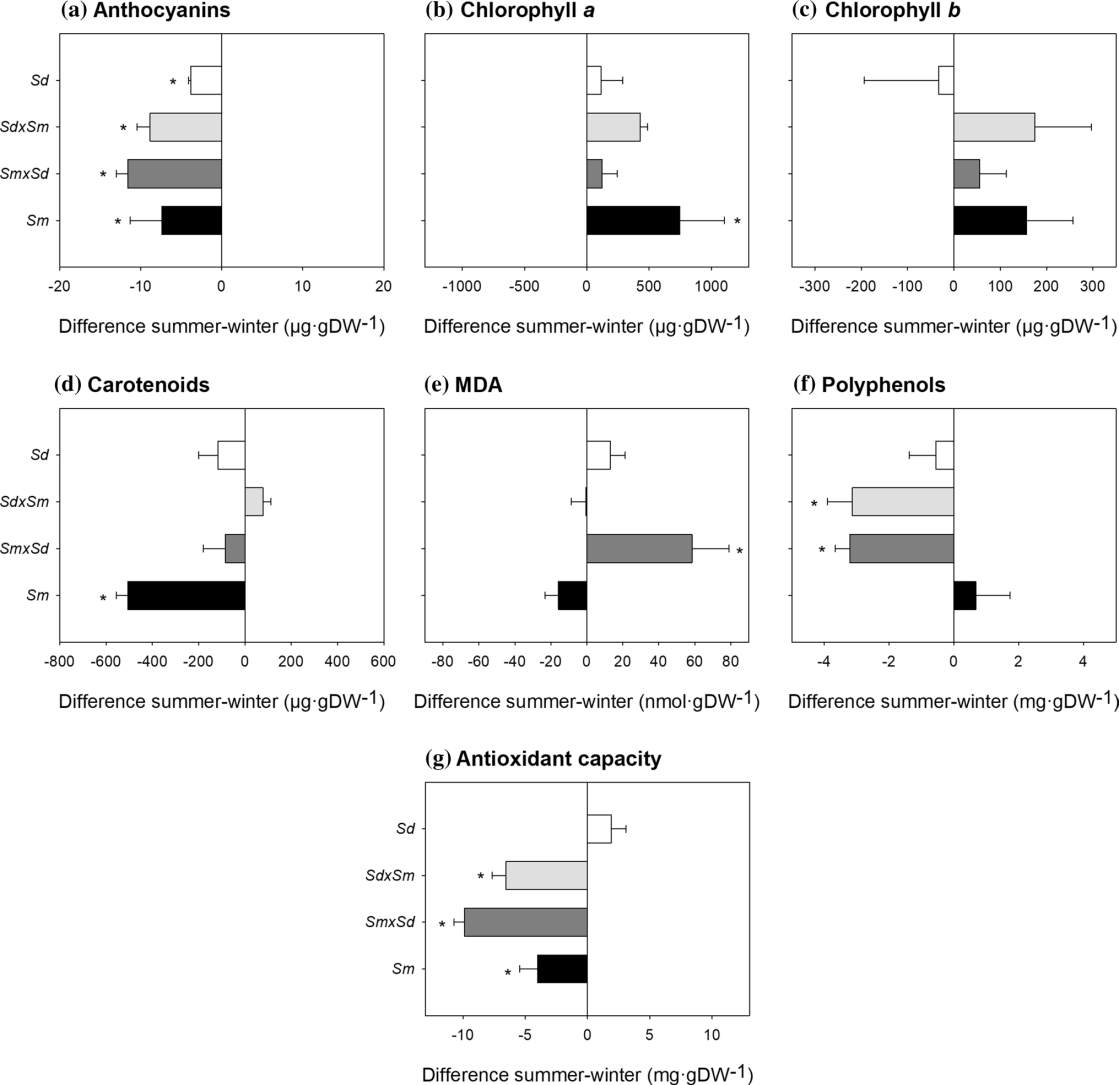
Seasonal differences in biochemical traits for *Spartina maritima* (*Sm*, black column), *Spartina densiflora* (*Sd*, white column) and their two reciprocal hybrids, *S. maritima* × *densiflora* (*Smxd*, dark grey column) and *S. densiflora* × *maritima* (*Sdxm*, light grey column). Values are means ± SE (*n* = 5). Negative values indicate higher magnitudes in winter and positive values indicate higher magnitudes in summer. Asterisks denote seasonal significance at *P* < 0.05 (GLM)

The four *Spartina* taxa exhibited higher anthocyanin concentration in winter than in summer, with *S. densiflora* showing a lower increase than the hybrids (Fig. 1A). *Spartina densiflora* and *S. densiflora* × *maritima* presented lower anthocyanin concentrations than *S. maritima* and *S. maritima* × *densiflora* (Table S1). *Spartina maritima* presented higher Chl *a* concentration and lower carotenoids concentration in summer than in winter (Fig. 1B, D). *Spartina maritima* accumulated more carotenoids in winter than *S. densiflora* and *S. densiflora* × *maritima* (Table S1). Chl *b* concentration did not change significantly between seasons for any taxa (Fig. 1C).

### MDA, antioxidant capacity and polyphenols

The hybrid *S. maritima* × *densiflora* was the only taxon that presented a higher (+ 68%) MDA concentration in summer than in winter (Fig. 1E). *Spartina maritima* × *densiflora* accumulated less MDA than *S. maritima* in winter, with *S. densiflora* and *S. densiflora* × *maritima* presenting intermediate values (Table S1). Both hybrids produced a higher (+ 35%) amount of polyphenols (Fig. 1F) and had greater (+ 47%) total antioxidant capacity in winter compared to summer. *Spartina maritima* also exhibited higher (+ 24%) total antioxidant capacity in winter than in summer (Fig. 1G). In winter, *S. maritima* accumulated more polyphenols than *S. densiflora* × *maritima* and *S. densiflora*. Total antioxidant capacity was lower for *S. densiflora* in winter and for *S. maritima* × *densiflora* in summer than for the other three taxa (Table S1).

### Growth and photosynthesis

**Fig. 2:**
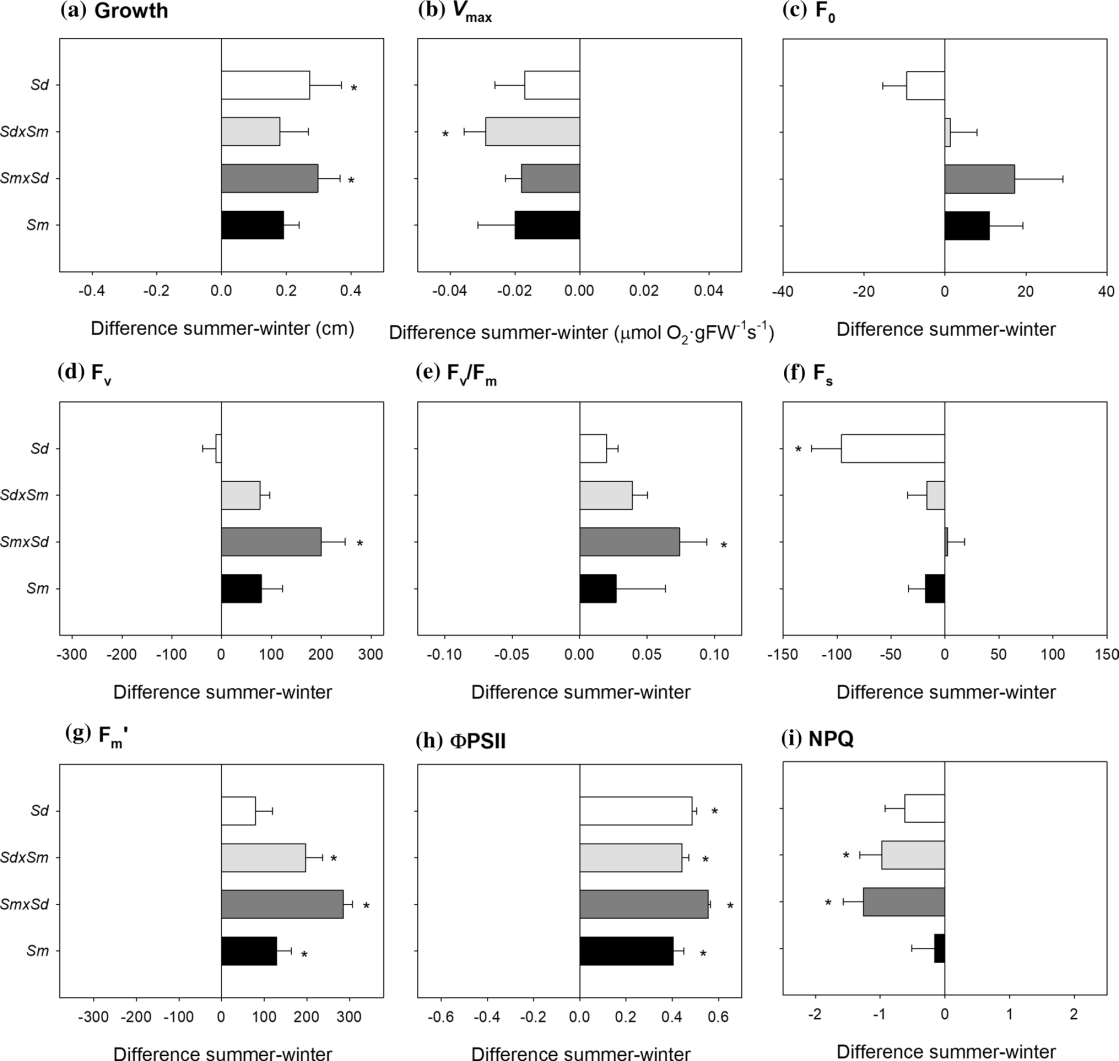
Seasonal differences in apical leaf growth (**A**) and *V*_max_ (**B**), and F_0_ (**C**), F_v_ (**D**), F_v_/F_m_ (**E**), F_s_ (**F**), F_m_’ (**G**), ΦPSII (**H**) and NPQ (**I**) at sunrise for *Spartina maritima* (*Sm*, black column), *Spartina densiflora* (*Sd*, white column) and their two reciprocal hybrids, *S. maritima* × *densiflora* (*Smxd*, dark grey) and *S. densiflora* × *maritima* (*Sdxm*, light grey). Values are means ± SE (*n* = 10). Negative values indicate higher magnitudes in winter, and positive values indicate higher magnitudes in summer. Asterisks denote seasonal significance at *P* < 0.05 (GLM)

Apical leaf growth was 38% higher in summer than in winter. This seasonal change was recorded for every taxon, being significant for *S. densiflora* and *S. maritima* × *densiflora* (Fig. 2A). *V*_max_ tended to be higher in winter than in summer for every taxon, although this seasonal variation was only significant for *S. densiflora* × *maritima* (Fig. 2B). *Spartina maritima* presented higher *V*_max_, but grew less than the other three taxa (Table S1).

### Chlorophyll fluorescence

*Spartina maritima* × *densiflora* was the only taxon showing higher (+ 9%) F_v_/F_m_ at sunrise in summer than in winter (Fig. 2E), through a marked increase (+ 46%) in F_v_ (Fig. 2D). ΦPSII at sunrise was markedly higher (+ 67%) in summer than in winter for all taxa (Fig. 2H) due to higher F_m_’ (Fig. 2G), except for *S. densiflora* that showed higher (+ 107%) F_s_ in winter than summer (Fig. 2F). Both hybrids presented higher ΦPSII at sunrise than *S. maritima* in summer. Additionally, the two hybrids showed higher NPQ at sunrise in winter than in summer, with *S. maritima* × *densiflora* exhibiting the greatest (+ 355%) seasonal increase (Fig. 2I) (Table S1).

**Fig. 3:**
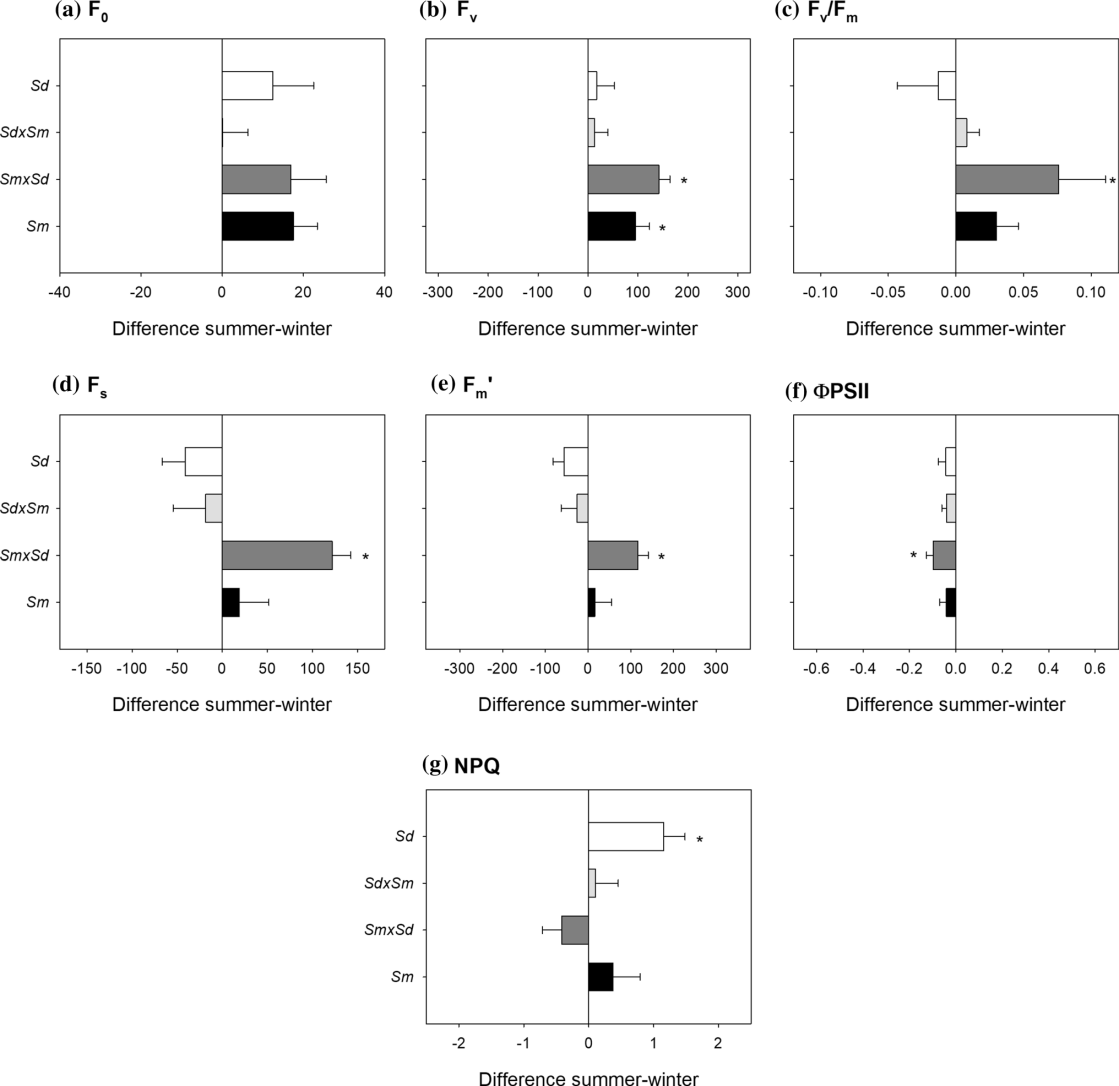
Seasonal differences in F_0_ (**A**), F_v_ (**B**), F_v_/F_m_ (**C**), F_s_ (**D**), F_m_’ (**E**), ΦPSII (**F**) and NPQ (**G**) at noon for *Spartina maritima* (*Sm*, black column), *Spartina densiflora* (*Sd*, white column) and their two reciprocal hybrids, *S. maritima* × *densiflora* (*Smxd*, dark grey column) and *S. densiflora* × *maritima* (*Sdxm*, light grey column). Values are means ± SE (*n* = 10). Negative values indicate higher magnitudes in winter, and positive values indicate higher magnitudes in summer. Asterisks denote seasonal significance at *P* < 0.05 (GLM)

When Chl fluorescence measurements were recorded at noon, *S. maritima* × *densiflora* exhibited the majority of the seasonal variations. This hybrid showed higher (+ 9%) F_v_/F_m_ [associated with increased (+ 46%) F_v_], and lower (− 229%) ΦPSII (with increased F_s_ and F_m_’ values) in summer than in winter (Fig. 3B–F). *S. densiflora* was the only taxon exhibiting higher (+ 59%) NPQ in summer than in winter (Fig. 3G), being higher than that recorded for *S. maritima* × *densiflora* (Table S1).

### Relationships between environmental matrix and plant responses

**Fig. 4:**
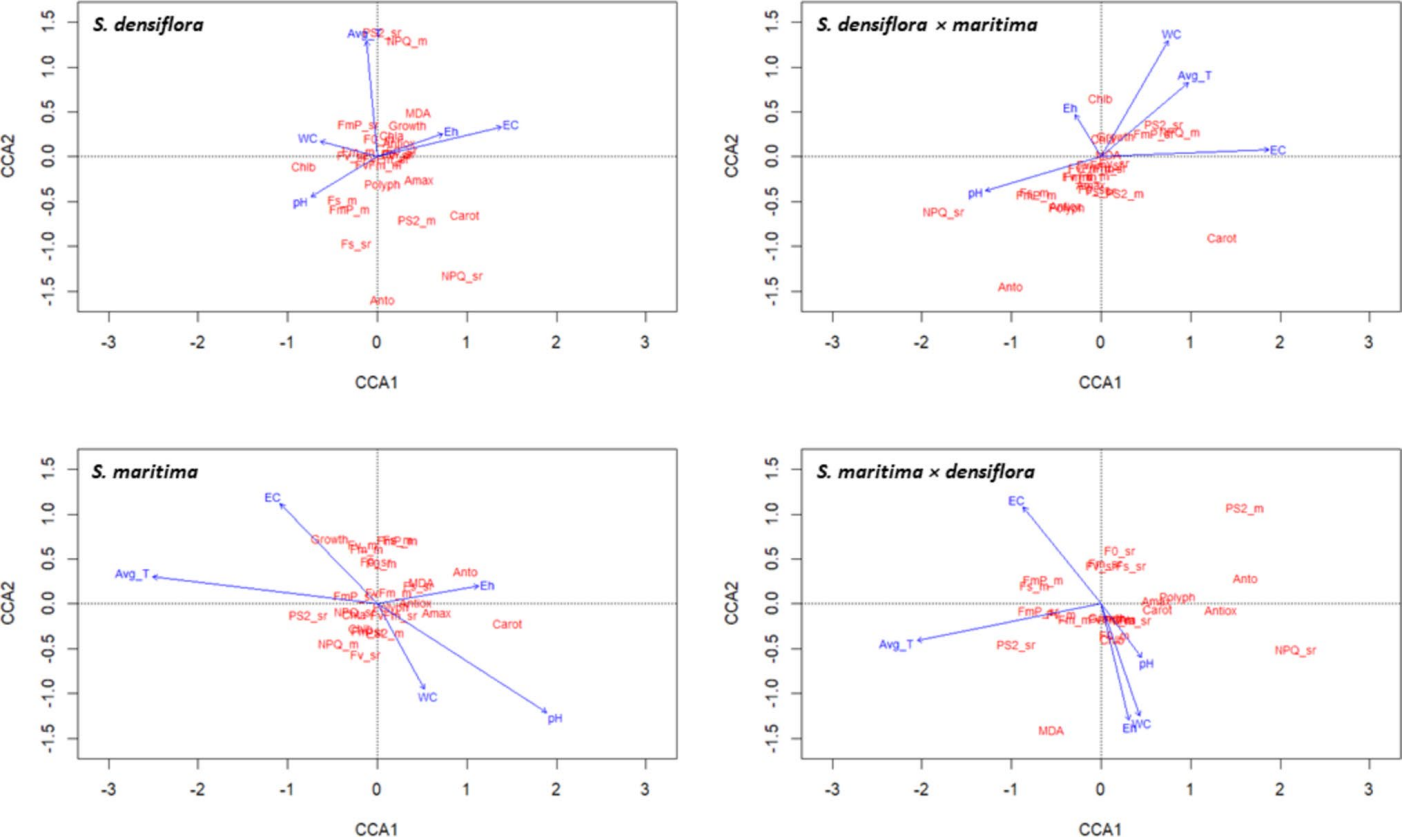
Ordination diagram of a Canonical Correspondence Analysis (CCA) with plant traits (red) and environmental variables (blue arrows) recorded in winter and summer for native *Spartina maritima*, alien *S. densiflora* and their reciprocal hybrids, *S. maritima* × *densiflora* and *S. densiflora* × *maritima* (*n* = 5 for each plant trait). Plant traits: Anthocyanins, total anthocyanin concentration; Antiox, total antioxidant capacity; Carotenoids, total carotenoid concentration; Chl*a*, chlorophyll *a* concentration; Chl*b*, chlorophyll *b* concentration; F_v_/F_m__sr, maximum quantum efficiency of PSII at sunrise; F_v_/F_m__m, maximum quantum efficiency of PSII at noon; Growth, apical leaf growth rate; MDA, malondialdehyde concentration; Polyphenols, total polyphenol concentration; NPQ_m, non-photochemical quenching at noon; NPQ_sr, non-photochemical quenching at sunrise; *V*_max_, Maximal rate of photosynthetic oxygen evolution; Φ_PSII__sr, quantum efficiency of PSII at sunrise; Φ_PSII__m, quantum efficiency of PSII at noon; F_0_, F_m_, F_v_, F_s_ and F_m_’ at sunrise and at noon have been removed from the plots so they can be seen more clearly. Environmental variables: Avg_T, average daily air temperature; EC, sediment electrical conductivity; Eh, sediment redox potential; pH, sediment pH; WC, sediment water content

Environmental conditions clearly distinguished plant trait responses for the four *Spartina* taxa (CCA ordination, Fig. 4, Tables S2–S5). For *S. maritima*, the first axis of the CCA explained 48% of the total variance in the relationships between plant traits and the environment and negatively correlated to average mean daily air temperature. In relation to plant traits, Axis 1 was positively correlated to carotenoid and anthocyanin concentrations and *V*_max_, and negatively to Φ_PSII_ at sunrise. Axis 2 explained 13% of the total variance and was mostly negatively correlated to sediment pH and WC, and positively to sediment EC and apical leaf growth rate (Fig. 4, Table S2).

Regarding *S*. *maritima* × *densiflora*, Axis 1 explained 57% of the total variance and correlated positively to anthocyanin concentration, total antioxidant capacity, Φ_PSII_ at noon and NPQ at sunrise and noon, and negatively to average mean daily air temperature and Φ_PSII_ at sunrise. Axis 2 explained 20% of the total variance and was mostly negatively correlated to sediment Eh and WC, and NPQ at noon (Fig. 4, Tables S3).

For *S. densiflora*, Axis 1 explained 39% of the total variance and was mostly and positively correlated with sediment EC, carotenoid concentration and NPQ at sunrise. Axis 2 explained 15% of the total variance and was positively correlated to average mean daily air temperature and Φ_PSII_ at sunrise, and negatively to anthocyanin concentration and NPQ at sunrise (Fig. 4, Tables S4).

In the case of *S. densiflora* × *maritima*, Axis 1 explained 36% of the total variance and was positively correlated with sediment EC, average daily temperature and carotenoid concentration, and negatively to NPQ at sunrise. Axis 2 explained 25% of the total variance and was positively correlated to sediment WC and negatively to anthocyanin and carotenoid concentrations (Fig. 4, Tables S5).

### Inheritance mechanisms of plant responses

**Fig. 5:**
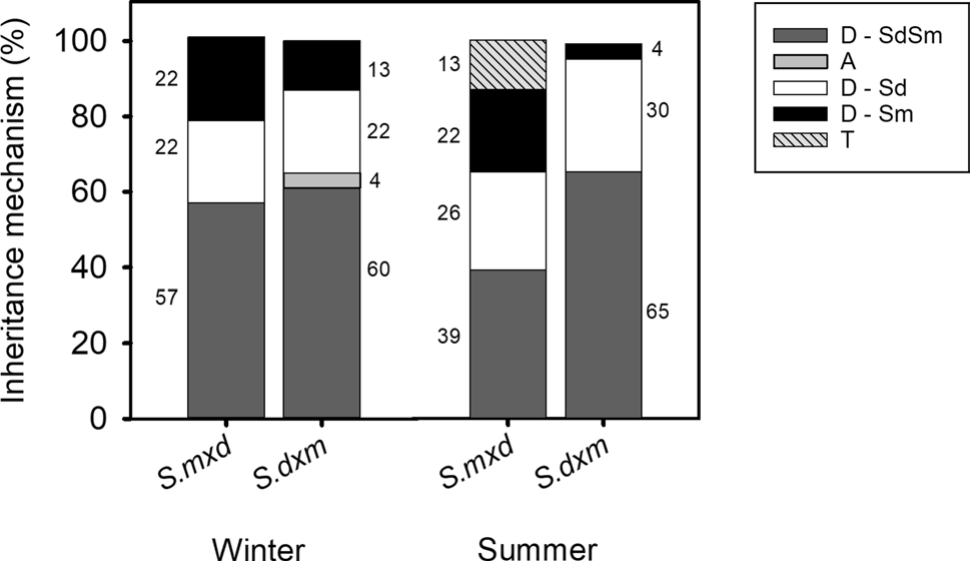
Percentage of inheritance mechanism of the measured traits in the reciprocal hybrids *S. maritima* × *densiflora* and *S. densiflora* × *maritima* in relation to their parents in winter and summer. D, parental dominance; Sm, *S. maritima*; Sd, *S. densiflora* (D-Sm = black, D-Sd = white, D-Sm,Sd = dark gray); A, parental additivity (light gray); T, transgressive (barred)

*Spartina maritima* × *densiflora* presented more traits dominated by *S. maritima* than *S. densiflora* × *maritima*. Both hybrids exhibited similar inheritance profiles, with a predominance of traits similar to both parental species (39–61%), followed by traits dominated by *S. densiflora* (22–30%) and, lastly, by *S. maritima* (13–22%). However, in winter, *S. densiflora* × *maritima* showed intermediate polyphenols concentrations to those exhibited by the parental species, which was the only recorded additive response (Fig. 5).

In relation to transgressive responses, *S. maritima* × *densiflora* in summer displayed 13% of transgressive traits related to radiation interaction (the lowest antioxidant capacity, and the lowest F_m_’ and highest F_v_ at sunrise). Additionally, the hybrids exhibited distinct seasonal changes that were unique compared to their parental species. *Spartina maritima* × *densiflora* was the only taxon showing lower MDA concentration, F_v_/F_m_, F_v_ and F_m_’ at sunrise and noon, and F_s_ at noon in winter than in summer, and higher Φ_PSII_ at noon in winter than in summer. *Spartina densiflora* × *maritima* was the only taxon presenting significantly higher *V*_max_ in winter than in summer. Finally, both hybrids showed higher polyphenols concentration and NPQ at sunrise in winter than in summer (Table S6).

## Discussion

Our study shows varied and contrasted responses to seasonal environmental changes at biochemical and ecophysiological levels for the native cordgrass *Spartina maritima*, the alien invasive *S. densiflora*, and their reciprocal hybrids in salt marshes under Mediterranean climate. As hypothesized, all studied *Spartina* taxa exhibited high tolerance to changing environmental conditions through acclimation of various functional traits. We identified only four common responses to seasonal environmental changes shared by all taxa, with no variation among taxa or due to the interaction between taxa and season. These responses were related to maximum and effective PSII quantum efficiency at noon and NPQ at sunrise. The hybrid *S. maritima* × *densiflora* was the most responsive taxon to seasonal changes, whereas *S. densiflora* showed the lowest number of seasonal acclimation responses (Table S6). As hypothesized, transgressivity and maternal effects played a relevant role in the response to seasonal environmental changes by the reciprocal hybrids.

Every taxon showed higher anthocyanin concentrations in the winter cold snap than in the summer heat wave, confirming the role of anthocyanin accumulation as a protective mechanism against cold stress. Anthocyanins are known to mitigate oxidative stress and provide photo-protection under low-temperature conditions (Shi et al. 2023). In contrast to the recorded seasonal change in anthocyanin concentration, Chl *b* concentration did not change seasonally in each taxon. This result pointed to Chl *b* playing a constant role in the light-harvesting complex and maintaining at a steady level to ensure the stability of the photosynthetic apparatus regardless of seasonal changes (Hoober and Eggink 2001). Another common response shared by every taxon was that Φ_PSII_ at sunrise was lower in winter than in summer, which would reflect the photosynthetic thermal stress related to low temperatures and relatively high radiation levels during sunny winter mornings (Doweler et al. 2021).

### Responses of the *Spartina* hybrids to environmental seasonal changes

The hybrid *S. maritima* × *densiflora* showed the highest number of seasonal-changing traits (16) followed by *S. densiflora* × *maritima* (7) (Table S6). Both hybrids accumulated more polyphenols and presented higher total antioxidant capacity, together with *S. maritima*, in the winter cold snap than in the summer heat wave. In addition, *S. maritima* × *densiflora* accumulated less MDA in winter, yielding a lower concentration than *S. maritima*. These results reflected the increasing ability of the hybrids for protection against oxidative stress in winter conditions (Król et al. 2015). Additionally, *S. maritima* × *densiflora* was the only taxon presenting higher NPQ at sunrise in winter than in summer, reflecting increasing levels of photo-protection through energy release by heat dissipation to deal with cold stress (Homayounfar et al. 2024). Despite this photo-protection mechanism, *S. maritima* × *densiflora* showed lower F_v_/F_m_ at sunrise and noon in winter than in summer, due to decreasing F_v_ values, which pointed to permanent damages to the photosynthetic apparatus related to cold stress (Maxwell and Johnson 2000; Murchie and Lawson 2013). At the same time, *S. maritima* × *densiflora* showed much higher ΦPSII at noon in winter than in summer, and both hybrids presented higher ΦPSII at sunrise than *S. maritima* in summer. These results suggested that the hybrids, especially *S. maritima* × *densiflora*, may have up-regulated photorespiration and/or utilizing cyclic electron flow around PSI (photo-phosphorylation) as supplementary photo-protective mechanisms (Popova et al. 2022). Both pathways can result in an increased consumption of reducing equivalents and can therefore serve as sinks for excess excitation energy. In addition, cyclic electron flow promotes lumenal acidification, favouring photoprotection for the photosynthetic apparatus (Huang et al. 2017). Furthermore, *S. maritima* × *densiflora* accumulated less Chl *b* than the other taxa, with similar values of Chl *a* in *S. densiflora* and *S. densiflora* × *maritima*. All these biochemical and ecophysiological acclimation responses to cold stress were reflected in higher *V*_max_ in winter than in summer, especially for *S. densiflora* × *maritima*. In general, all these results characterized both hybrids as more responsive taxa to seasonal environmental changes than their parental species. Consequently, *S. maritima* × *densiflora* presented 13% of transgressive responses in summer. In the same direction, previous studies also identified *S. maritima* × *densiflora* as a hybrid showing key transgressive functional traits in response to salinity variations in controlled conditions related to phosphoenolpyruvate carboxylase (PEPC) enzyme and to Chl fluorescence (NPQ at sunrise at 10 ppt and Φ_PSII_ at noon at 20–40 ppt; the same traits identified as transgressive in our study) (Gallego-Tévar et al. 2018a, 2020b). The relatively high number of transgressive traits recorded for *S. maritima* × *densiflora* (2n = 95) in relation to *S. densiflora* × *maritima* (2n = 65) may be related to their different ploidy levels that can induce gene dosage and allometric effects (Zhang et al. 2019).

The recorded functional traits for *Spartina densiflora* and *S. densiflora* × *maritima* responded mostly to seasonal variations in sediment salinity (recorded as EC) and these taxa presented lower anthocyanin concentrations than *S. maritima* and *S. maritima* × *densiflora* that responded mostly to air temperature changes. Moreover, *S. maritima* dominated the hereditary transfer of functional traits in *S. maritima* × *densiflora* compared to *S. densiflora* × *maritima*. These results point to maternal effects playing an important role in transgenerational plasticity in response to seasonal environmental changes by the hybrids (Iida et al. 2013).

### Responses of parental species to environmental seasonal changes

Native *S. maritima* presented 7 functional traits that changed seasonally mostly in response to air temperature. This species, together with *S. maritima* × *densiflora*, accumulated more polyphenols than the other two taxa in the winter cold snap, reflecting its ability to protect itself against oxidative stress in winter conditions of low temperatures and high radiation (Páldi et al. 2014). Duarte et al. (2013) demonstrated that *S. maritima* accumulates relatively high concentrations of phenolic compounds in response to metal pollution. This suggests that the biochemical defenses of *S. maritima* could serve as effective biomarkers in studies assessing estuarine sediment quality. Moreover, *S. maritima* was the only taxon accumulating more carotenoids in winter than in summer in relation to decreasing temperatures. These results reflect enhanced antioxidant and photoprotection capacities during cold stress (Wang et al. 2024), added to those coming from the above-mentioned high anthocyanin accumulation, as reflected in increased total antioxidant capacity in winter. All these acclimation responses to cold stress in *S. maritima* yielded similar apical leaf growth rates in winter and summer. Moreover, *S. maritima* was the taxon showing the highest *V*_max_ values, as reported by Gallego-Tévar et al. (2020b) at 20 ppt salinity, which were positively related to decreasing air temperatures. Duarte et al. (2015) described *S. maritima* as a well-adapted species to Mediterranean winter conditions. On the other hand, *S. maritima* was the only taxon accumulating more Chl *a* in summer than in winter, yielding higher concentrations than *S. maritima* × *densiflora*. This accumulation of Chl *a* indicated increased photosynthetic activity during the summer, likely driven by higher temperatures and radiation levels (Li et al. 2022).

Alien *S. densiflora* was the taxon presenting the lower number of seasonal changes (5), responding mostly to sediment salinity. Previous studies have characterized the photosynthetic metabolism of* S. densiflora* as highly sensitive to salinity (Castillo et al. 2005; Gallego-Tévar et al. 2020b, c). *Spartina densiflora* has been described as showing high levels of phenotypic plasticity along a wide latitudinal gradient in its introduced range in North America in summer conditions, where spatial salinity variations (Δ13.6 mS^−1^) were higher than the recorded seasonal variation in our study (Δ2.5 mS cm^−1^) (Castillo et al. 2018). *Spartina densiflora* was the only taxon that showed lower NPQ at noon in the winter cold snap than in the summer heat wave. Moreover, *S. densiflora* did not increase its total antioxidant capacity in winter. Therefore, *S. densiflora* showed lower values of polyphenols and antioxidant capacity than the other three taxa in winter. Additionally, *S. densiflora* accumulated less anthocyanins in winter in relation to summer than both hybrids. As a result of this relatively low acclimation to winter conditions, *S. densiflora* showed a lower apical leaf growth rate in winter than in summer. However, we did not record permanent damages to the photosynthetic apparatus for *S. densiflora*. Previous studies have reported that plants pre-acclimated to salinity stress show high tolerances to thermal stress (Liu et al. 2023) since both stresses share some common responses such as accumulation of certain osmolytes (Walker and Lutts 2014). Under conditions of increasing salinity, the halophytic species *Zostera japonica* Asch. and Graebn displayed an increased capacity to tolerate chronic exposure to elevated temperatures (35 ºC) (Kaldy and Shafer 2013).

### Seasonal responses and climate change

In view of our results, both hybrids appear as taxa with high acclimation capacity in a wide range of functional traits that protect their photosynthetic apparatus from thermal stress. This would allow them to acclimate adequately to the increasingly frequent cold and heat waves in the Mediterranean Basin in the present scenario of climate change (D’Errico et al. 2022). According to the tolerance to changing meteorological conditions, both *Spartina* hybrids would be able to expand their geographical distribution ranges from the Gulf of Cadiz to salt marshes northern along the European Atlantic Coast. Even so, the hybrids have limited dispersion capacities due to their sterility (Castillo et al. 2010). It is crucial to monitor the studied hybrid taxa for chromosome doubling, as this could result in fertile allopolyploid species with a potentially significant impact on local biodiversity. Given the current context of climate change, further field ecophysiological studies are needed to better understand the ecological role of these hybrid taxa and their tolerance to extreme weather events compared to their parental species. Hybrids exhibiting transgressive traits may be particularly valuable for climate change adaptation in agriculture and as biotools in ecological restoration projects, especially in situations where the environmental tolerance of their parental species is exceeded by changing environmental conditions.

Native *S. maritima* appears as taxa with high acclimation capacity to thermal stress. This result agrees with *S. maritima* inhabiting northern European salt marshes where it is exposed to lower temperatures than in our study site (Marchant and Goodman 1969). In contrast to the other three taxa, alien *S. densiflora* showed relatively less acclimation responses to changing meteorological conditions without presenting permanent damages to its photosynthetic apparatus. *Spartina densiflora* has invaded marshes as north as British Columbia on the Atlantic Coast of North America (Castillo et al. 2014). Therefore, an early detection network of *S. densiflora* invasion should be established along the Atlantic Coast of the Iberian Peninsula to eradicate it as soon as possible after its colonization.

Following our results, the four studied *Spartina* taxa seem to be adequately preadapted to the present scenario of climate change. Nevertheless, future heat waves may expose the studied *Spartina* taxa to higher temperatures than those recorded in our study, compromising their photosynthetic capacity. In this regard, Duarte et al. (2016) reported that *S. maritima* presents high energy losses and decrease in the enzymatic defenses under heat stress at higher temperatures (ca. 40 ºC) than those recorded in our study (max. ca. 32 ºC). Crosby et al. (2017) showed that transplants of *Spartina alterniflora* Loisel from northern populations died when moved south along the Atlantic Coast of North America, suggesting that northern plants may be severely stressed by future warming.

Our study investigated the responses of four *Spartina* taxa to extreme meteorological conditions, the frequency of which is increasing under the current climate change scenario. Additional field research is required to assess the seasonal responses of these *Spartina* taxa under moderate meteorological conditions, including winter conditions, given their lack of dormancy in the study area.

## Conclusions

Closely-related *Spartina* taxa responded in contrasted ways to marked seasonal environmental changes. Both hybrid taxa, especially *S. maritima* × *densiflora*, presented transgressive responses that enabled them to show a wider range of acclimation responses to air temperature than their parental species. Native *S. maritima* also showed a relatively high acclimation capacity to seasonal meteorological changes. In contrast, alien *S. densiflora* presented a relatively low acclimation response to seasonal environmental changes, responding mostly to sediment salinity. Therefore, *S. densiflora* and both hybrids would be able to expand their geographical distribution ranges to northern European salt marshes and the four studied *Spartina* taxa seem to be well adapted to occurring cold and heat waves in the Gulf of Cadiz.

## Supplementary Information

Below is the link to the electronic supplementary material.

Supplementary file1 (DOCX 2689 KB)

## Data Availability

The data sets generated and analyzed in this study are available from the corresponding author on reasonable request.

## Abbreviations

EC: Electrical conductivity
Eh: Redox potential
F_0_, F_m_, F_v_: Initial, maximal, and variable fluorescence
F_v_/F_m_: Maximum quantum efficiency of PSII
MDA: Malondialdehyde
NPQ: Non-photochemical quenching
WC: Water content
*V*_max_: Maximal rate of photosynthetic oxygen evolution
Φ_PSII_: Effective quantum efficiency of PSII
