# Supplementary material for: Ecophysiological and biochemical responses to cold and heat waves of native Spartina maritima, alien S. densiflora and their reciprocal hybrids
Source: Planta. 2025 Apr 1;261(5):99. doi: 10.1007/s00425-025-04675-4 (PMC11961512; doi:10.1007/s00425-025-04675-4)
Supplement: Supplementary file 1 — Supplementary file1 (DOCX 2689 KB) [file 425_2025_4675_MOESM1_ESM.docx]

**Supplemental information**

**Fig. S1** Location of the Guadiana Marshes in southwest Iberian Peninsula and aerial photograph showing the sampling area. Green area, low marsh where *Spartina maritima* and *S. maritima × densiflora* were sampled; red area, middle marsh where *S. densiflora* and *S. densiflora × maritima* were sampled


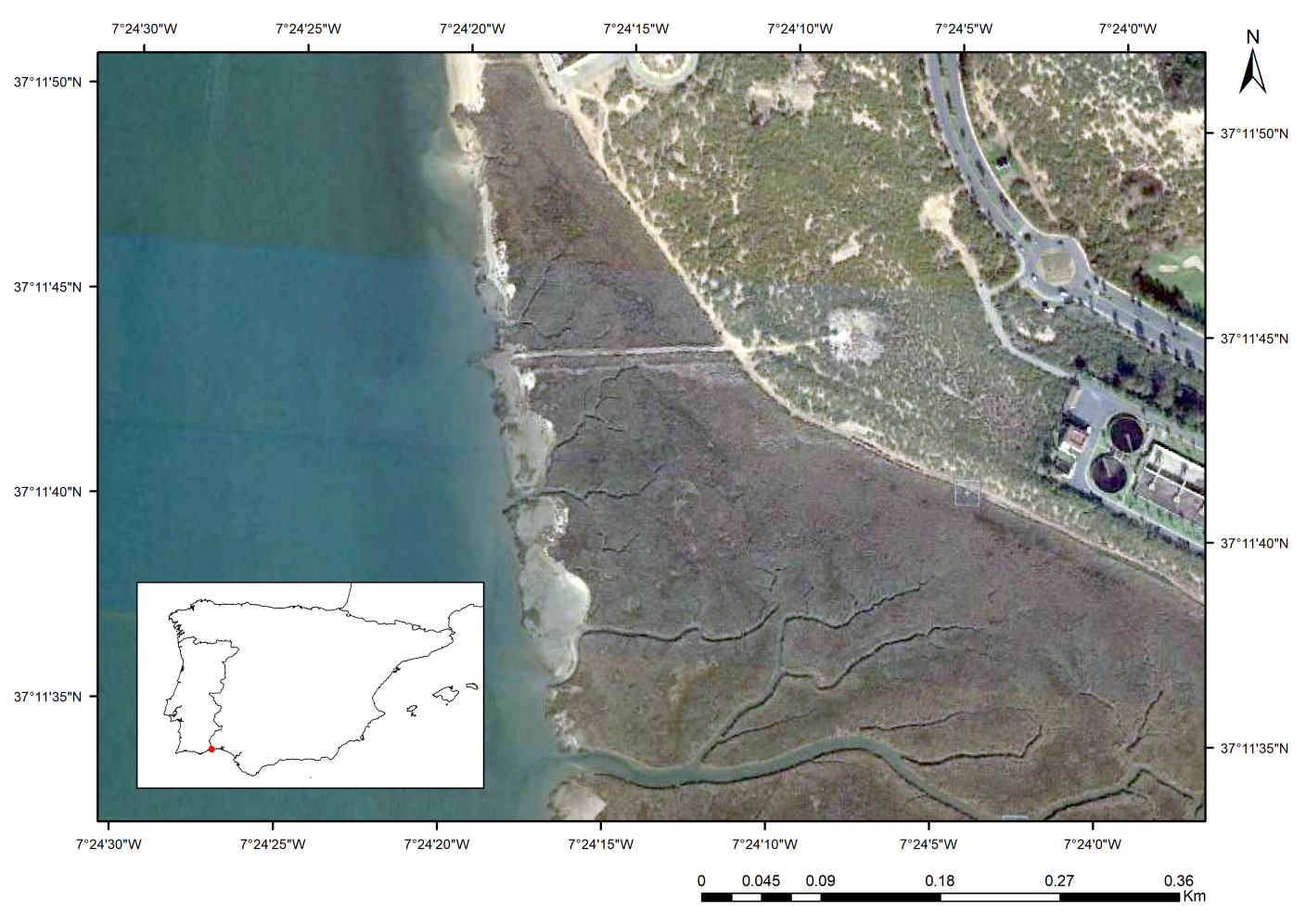


**Fig. S2** Climograph illustrating the monthly variation in average, maximum and minimum temperatures, and precipitation for the study area in the sampling year 2021 (Data source: AEMET, 2023 for the station 4549Y, Ayamonte, Spain)


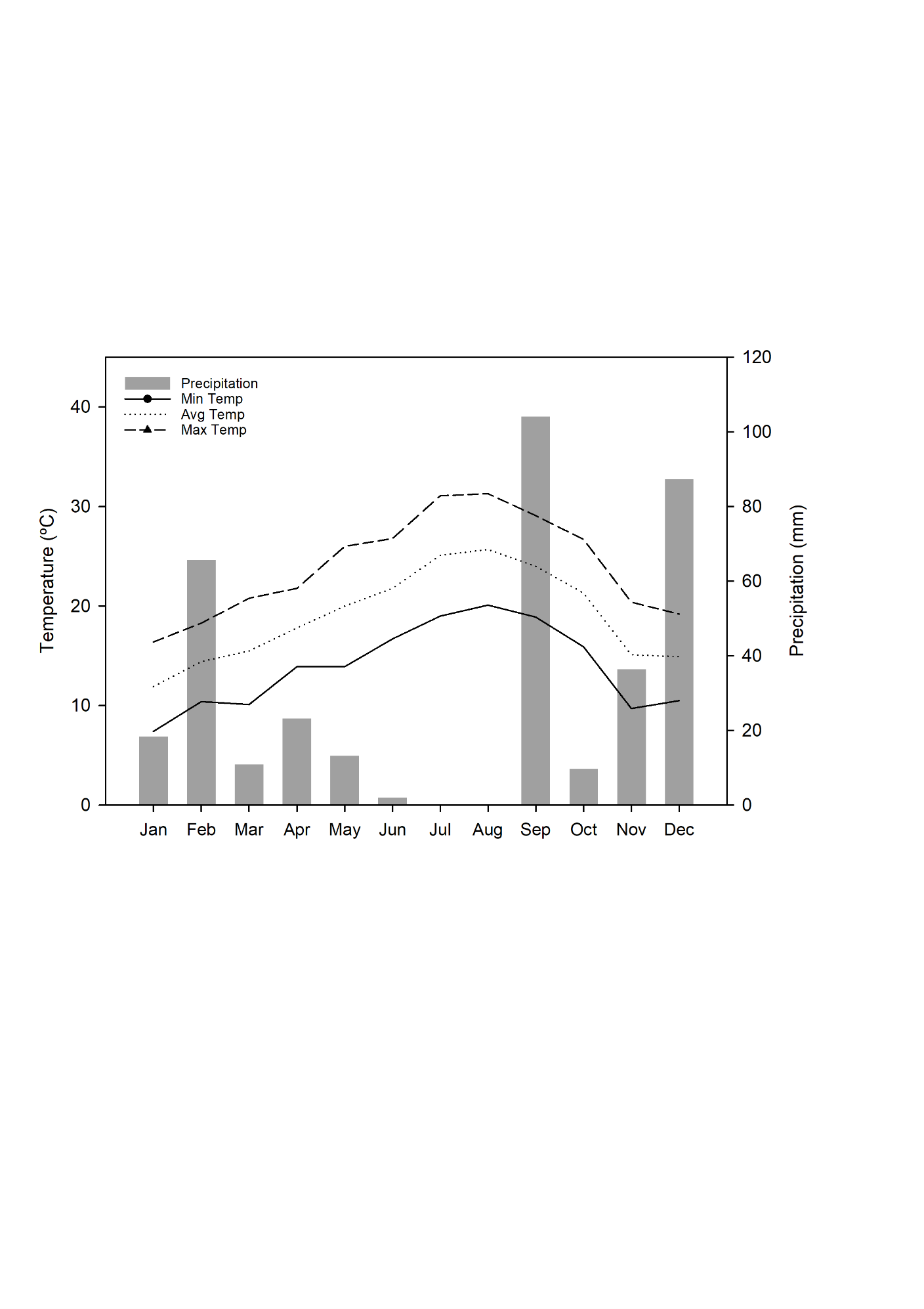


**Table S1** Biochemical and ecophysiological and growth-related traits measured in individuals of *Spartina maritima*, *Spartina densiflora* and their reciprocal hybrids in the Guadiana Marshes (Southwest Iberian Peninsula) in a cold snap in winter and in a heat wave in summer. Values are arithmetic mean ± SE (*n* = 5-10). Different letters indicate significant differences between taxa for the same plant trait and season

|  | *S. maritima* | *S. densiflora* | *S. maritima × densiflora* | *S. densiflora* *× maritima* |
| --- | --- | --- | --- | --- |
| Winter |  |  |  |  |
| Anthocyanins (µg g DW^-1^) | 14 ± 4^a^ | 5 ± 0^b^ | 16 ± 1^a^ | 10 ± 2^ab^ |
| Chl *a* (µg g DW^-1^) | 1606 ± 113 | 1369 ± 65 | 1046 ± 63 | 1297 ± 81 |
| Chl *b* (µg g DW^-1^) | 492 ± 43^a^ | 500 ± 47^a^ | 242 ± 18^b^ | 406 ± 39^a^ |
| Carotenoids (µg g DW^-1^) | 670 ± 30^a^ | 335 ± 24^bc^ | 437 ± 23^ab^ | 149 ± 71^c^ |
| MDA (nmol g DW^-1^) | 70 ± 8^a^ | 40 ± 3^ab^ | 28 ± 6^b^ | 42 ± 8^ab^ |
| Polyphenols (mg g DW^-1^) | 11 ± 0^a^ | 4 ± 0^c^ | 9 ± 0^ab^ | 8 ± 0^b^ |
| Antioxidant capacity (mg g DW^-1^) | 17 ± 1^a^ | 9 ± 1^b^ | 17 ± 1^a^ | 17 ± 1^a^ |
| Apical leaf growth (cm) | 0.17 ± 0.01^a^ | 0.52 ± 0.03^b^ | 0.50 ± 0.07^b^ | 0.46 ± 0.07^b^ |
| *V*_max_ (μmol O_2_ s^-1^ g^-1^ FW^-1^) | 0.090 ± 0.008^a^ | 0.067 ± 0.005^b^ | 0.068 ± 0.005^b^ | 0.076 ± 0.006^b^ |
| F_0_ at sunrise | 63 ± 4 | 72 ± 4 | 73 ± 10 | 68 ± 4 |
| F_v_ at sunrise | 187 ± 20^a^ | 305 ± 24^b^ | 238 ± 42^ab^ | 259 ± 22^ab^ |
| F_v_/F_m_ at sunrise | 0.743 ± 0.017 | 0.807 ± 0.007 | 0.755 ± 0.019 | 0.789 ± 0.007 |
| F_s_ at sunrise | 113 ± 13^a^ | 185 ± 29^b^ | 110 ± 17^a^ | 104 ± 17^a^ |
| F_m_’ at sunrise | 146 ± 18 | 232 ± 32 | 133 ± 20 | 149 ± 23 |
| ΦPSII at sunrise | 0.216 ± 0.032^ab^ | 0.224 ± 0.021^ab^ | 0.176 ± 0.013^a^ | 0.304 ± 0.034^b^ |
| NPQ at sunrise | 0.85 ± 0.17 | 0.96 ± 0.29 | 1.61 ± 0.28 | 1.52 ± 0.25 |
| F_0_ at noon | 68 ± 6 | 62 ± 5 | 64 ± 9 | 75 ± 5 |
| F_v_ at noon | 197 ± 19^a^ | 226 ± 23^ab^ | 167 ± 23^a^ | 292 ± 24^b^ |
| F_v_/F_m_ at noon | 0.740 ± 0.016 | 0.776 ± 0.21 | 0.719 ± 0.029 | 0.793 ± 0.007 |
| F_s_ at noon | 141 ± 26^ab^ | 151 ± 18^ab^ | 97 ± 17^a^ | 196 ± 36^b^ |
| F_m_’ at noon | 162 ± 29^ab^ | 176 ± 19^ab^ | 113 ± 20^a^ | 220 ± 38^b^ |
| ΦPSII at noon | 0.138 ± 0.021 | 0.152 ± 0.015 | 0.139 ± 0.023 | 0.126 ± 0.015 |
| NPQ at noon | 1.03 ± 0.26 | 0.81 ± 0.11 | 1.20 ± 0.23 | 1.00 ± 0.27 |
| Summer |  |  |  |  |
| Anthocyanins (µg g DW^-1^) | 7 ± 1^a^ | 2 ± 0^b^ | 4 ± 1^a^ | 1 ± 0^b^ |
| Chl *a* (µg g DW^-1^) | 2353 ± 284 | 1484 ± 180 | 1169 ± 116 | 1727 ± 110 |
| Chl *b* (µg g DW^-1^) | 649 ± 89^a^ | 467 ± 143^a^ | 298 ± 51^b^ | 582 ± 135^a^ |
| Carotenoids (µg g DW^-1^) | 164 ± 25 | 219 ± 75 | 352 ± 77 | 226 ± 55 |
| MDA (nmol g DW^-1^) | 54 ± 4^ab^ | 53 ± 9^ab^ | 86 ± 20^a^ | 41 ± 1^b^ |
| Polyphenols (mg g DW^-1^) | 12 ± 1 | 4 ± 1 | 6 ± 0 | 5 ± 1 |
| Antioxidant capacity (mg g DW^-1^) | 13 ± 1^a^ | 11 ± 1^a^ | 7 ± 0^b^ | 11 ± 1^a^ |
| Apical leaf growth (cm) | 0.36 ± 0.04^a^ | 0.79 ± 0.08^b^ | 0.80 ± 0.05^b^ | 0.64 ± 0.06^b^ |
| *V*_max_ (μmol O_2_ s^-1^ g^-1^ FW^-1^) | 0.070 ± 0.008^a^ | 0.051 ± 0.006^b^ | 0.050 ± 0.003^b^ | 0.046 ± 0.002^b^ |
| F_0_ at sunrise | 74 ± 7 | 62 ± 5 | 90 ± 5 | 69 ± 5 |
| F_v_ at sunrise | 267 ± 32^a^ | 293 ± 19^a^ | 438 ± 18^b^ | 336 ± 18^ab^ |
| F_v_/F_m_ at sunrise | 0.770 ± 0.30 | 0.826 ± 0.006 | 0.829 ± 0.007 | 0.829 ± 0.011 |
| F_s_ at sunrise | 95 ± 7 | 89 ± 4 | 113 ± 8 | 88 ± 7 |
| F_m_’ at sunrise | 276 ± 30^a^ | 312 ± 10^a^ | 419 ± 25^b^ | 345 ± 26^ab^ |
| ΦPSII at sunrise | 0.621 ± 0.041^a^ | 0.711 ± 0.015^ab^ | 0.731 ± 0.012^b^ | 0.745 ± 0.009^b^ |
| NPQ at sunrise | 0.69 ± 0.24 | 0.34 ± 0.03 | 0.35 ± 0.07 | 0.55 ± 0.18 |
| F_0_ at noon | 86 ± 6 | 74 ± 8 | 80 ± 5 | 75 ± 4 |
| F_v_ at noon | 292 ± 20 | 243 ± 27 | 309 ± 11 | 305 ± 11 |
| F_v_/F_m_ at noon | 0.769 ± 0.014 | 0.762 ± 0.020 | 0.794 ± 0.011 | 0.802 ± 0.007 |
| F_s_ at noon | 160 ± 19^ab^ | 110 ± 20^a^ | 219 ± 20^b^ | 178 ± 15^ab^ |
| F_m_’ at noon | 178 ± 23^ab^ | 120 ± 21^a^ | 229 ± 21^b^ | 195 ± 16^ab^ |
| ΦPSII at noon | 0.097 ± 0.023 | 0.110 ± 0.026 | 0.042 ± 0.008 | 0.087 ± 0.018 |
| NPQ at noon | 1.41 ± 0.27^ab^ | 1.97 ± 0.34^a^ | 0.79 ± 0.11^b^ | 1.10 ± 0.21^ab^ |

**Table S2** Eigenvalues, proportion of variance explained and regression coefficients in a Canonical Correspondence Analysis (CCA) for canonical axes against standardized variables for ordination of plant trait responses relative to environmental conditions in winter and summer for *Spartina maritima* in Guadiana salt marshes (Southwest Iberian Peninsula). Correlations with scores > ± 0.450 are marked in bold

| *Spartina maritima* | Axis 1 | Axis 2 | Axis 3 | Axis 4 | Axis 5 |
| --- | --- | --- | --- | --- | --- |
| Eigenvalues | 0.053 | 0.015 | 0.008 | 0.003 | 0.001 |
| Variance explained | 0.479 | 0.130 | 0.071 | 0.027 | 0.013 |
| Environmental variables |  |  |  |  |  |
| Average daily air temperature | **-0.968** | 0.162 | -0.108 | 0.135 | -0.082 |
| Sediment redox potential | 0.436 | 0.106 | 0.303 | -0.219 | **-0.812** |
| Sediment water content | 0.202 | **-0.509** | -0.591 | **-0.523** | -0.278 |
| Sediment pH | **0.729** | **-0.647** | -0.167 | -0.114 | -0.095 |
| Sediment electrical conductivity | -0.419 | **0.597** | **0.571** | -0.235 | 0.294 |
| Plant traits |  |  |  |  |  |
| Maximal rate of photosynthetic oxygen evolution | 0.322 | -0.033 | -0.075 | 0.149 | -0.094 |
| Total antioxidant capacity | 0.209 | 0.007 | -0.007 | -0.119 | 0.028 |
| Anthocyanin content | **0.476** | 0.128 | -0.300 | -0.253 | 0.029 |
| Carotenoid content | **0.702** | -0.073 | 0.043 | 0.004 | -0.018 |
| Chlorophyll *a* content | -0.120 | -0.040 | 0.060 | 0.009 | 0.000 |
| Chlorophyll *b* content | -0.085 | -0.094 | 0.048 | -0.057 | 0.020 |
| F_0_ at noon | 0.023 | 0.153 | 0.022 | -0.008 | -0.045 |
| F_0_ at sunrise | -0.005 | 0.160 | -0.125 | 0.050 | 0.107 |
| F_m_ at noon | -0.054 | 0.208 | -0.002 | -0.011 | -0.026 |
| F_m_ at sunrise | -0.049 | -0.110 | -0.180 | 0.073 | 0.015 |
| F_m_’ at noon | 0.106 | 0.242 | -0.021 | 0.108 | 0.008 |
| F_m_’ at sunrise | -0.119 | 0.028 | -0.190 | -0.126 | -0.076 |
| F_s_ at noon | 0.114 | 0.244 | 0.008 | 0.080 | 0.055 |
| F_s_ at sunrise | 0.220 | 0.068 | -0.140 | -0.154 | 0.166 |
| F_v_ at noon | -0.078 | 0.225 | -0.010 | -0.012 | -0.020 |
| F_v_ at sunrise | -0.063 | -0.200 | -0.198 | 0.081 | -0.016 |
| F_v_/F_m_ at noon | 0.063 | 0.040 | -0.033 | -0.027 | 0.026 |
| F_v_/F_m_ at sunrise | 0.089 | -0.046 | -0.029 | -0.021 | -0.013 |
| Apical leaf growth rate | -0.252 | 0.254 | -0.168 | 0.235 | -0.181 |
| MDA content | 0.240 | 0.085 | -0.052 | -0.052 | -0.101 |
| NPQ at noon | -0.206 | -0.159 | -0.035 | -0.072 | -0.057 |
| NPQ at sunrise | -0.118 | -0.034 | 0.254 | 0.100 | 0.100 |
| Polyphenol content | 0.076 | -0.014 | -0.080 | 0.047 | -0.059 |
| Φ_PSII_ at noon | 0.044 | -0.114 | -0.174 | 0.107 | -0.155 |
| Φ_PSII_ at sunrise | -0.370 | -0.046 | -0.050 | -0.019 | -0.185 |

**Table S3** Eigenvalues, proportion of variance explained and regression coefficients in a Canonical Correspondence Analysis (CCA) for canonical axes against standardized variables for ordination of plant trait responses relative to environmental conditions in winter and summer for *Spartina maritima × densiflora* in Guadiana salt marshes (Southwest Iberian Peninsula). Correlations with scores > ± 0.450 are marked in bold

| *Spartina maritima × densiflora* | Axis 1 | Axis 2 | Axis 3 | Axis 4 | Axis 5 |
| --- | --- | --- | --- | --- | --- |
| Eigenvalues | 0.031 | 0.011 | 0.007 | 0.005 | 0.001 |
| Variance explained | 0.571 | 0.200 | 0.125 | 0.090 | 0.014 |
| Environmental variables |  |  |  |  |  |
| Average daily air temperature | **-0.864** | -0.222 | 0.427 | -0.046 | -0.137 |
| Sediment redox potential | 0.131 | **-0.709** | **-0.506** | **0.470** | -0.049 |
| Sediment water content | 0.180 | **-0.684** | **0.614** | 0.347 | 0.038 |
| Sediment pH | 0.191 | -0.326 | 0.325 | **0.866** | -0.032 |
| Sediment electrical conductivity | -0.366 | **0.593** | -0.192 | -0.422 | **-0.547** |
| Plant traits |  |  |  |  |  |
| Maximal rate of photosynthetic oxygen evolution | 0.259 | 0.013 | -0.179 | -0.088 | 0.166 |
| Total antioxidant capacity | **0.562** | -0.020 | 0.020 | 0.044 | -0.025 |
| Anthocyanin content | **0.681** | 0.096 | 0.009 | 0.129 | 0.040 |
| Carotenoid content | 0.268 | -0.016 | -0.034 | -0.115 | 0.012 |
| Chlorophyll *a* content | 0.112 | -0.054 | 0.225 | 0.017 | 0.105 |
| Chlorophyll *b* content | 0.055 | -0.127 | -0.086 | -0.050 | 0.007 |
| F_0_ at noon | 0.064 | -0.117 | -0.162 | -0.021 | 0.080 |
| F_0_ at sunrise | 0.092 | 0.188 | 0.028 | 0.068 | 0.007 |
| F_m_ at noon | -0.123 | -0.060 | 0.073 | 0.014 | 0.001 |
| F_m_ at sunrise | 0.019 | 0.144 | 0.084 | 0.001 | -0.001 |
| F_m_’ at noon | -0.273 | 0.084 | -0.019 | -0.062 | 0.039 |
| F_m_’ at sunrise | -0.291 | -0.029 | -0.118 | -0.028 | 0.058 |
| F_s_ at noon | -0.312 | 0.061 | 0.134 | -0.117 | 0.001 |
| F_s_ at sunrise | 0.141 | 0.135 | 0.275 | -0.122 | -0.010 |
| F_v_ at noon | -0.184 | -0.041 | -0.381 | 0.037 | 0.020 |
| F_v_ at sunrise | 0.001 | 0.134 | -0.092 | -0.171 | 0.013 |
| F_v_/F_m_ at noon | 0.050 | -0.059 | -0.104 | -0.041 | -0.022 |
| F_v_/F_m_ at sunrise | 0.127 | -0.063 | -0.108 | 0.001 | -0.033 |
| Apical leaf growth rate | 0.032 | -0.047 | -0.082 | -0.009 | 0.004 |
| MDA content | -0.234 | **-0.455** | -0.103 | 0.035 | 0.023 |
| NPQ at noon | **0.574** | **-0.579** | -0.114 | 0.067 | 0.027 |
| NPQ at sunrise | **0.918** | -0.166 | 0.098 | 0.228 | 0.234 |
| Polyphenol content | 0.361 | 0.023 | -0.091 | -0.317 | 0.005 |
| Φ_PSII_ at noon | **0.679** | 0.344 | -0.012 | -0.094 | 0.146 |
| Φ_PSII_ at sunrise | -0.399 | -0.149 | 0.191 | -0.168 | 0.217 |

**Table S4** Eigenvalues, proportion of variance explained and regression coefficients in a Canonical Correspondence Analysis (CCA) for canonical axes against standardized variables for ordination of plant trait responses relative to environmental conditions in winter and summer for *Spartina densiflora* in Guadiana salt marshes (Southwest Iberian Peninsula). Correlations with scores > ± 0.450 are marked in bold

| *Spartina densiflora* | Axis 1 | Axis 2 | Axis 3 | Axis 4 | Axis 5 |
| --- | --- | --- | --- | --- | --- |
| Eigenvalues | 0.032 | 0.013 | 0.005 | 0.004 | 0.003 |
| Variance explained | 0.386 | 0.154 | 0.055 | 0.047 | 0.035 |
| Environmental variables |  |  |  |  |  |
| Average daily air temperature | -0.067 | **0.907** | 0.032 | -0.399 | 0.134 |
| Sediment redox potential | 0.405 | 0.179 | -0.425 | 0.713 | -0.337 |
| Sediment water content | -0.355 | 0.123 | **0.571** | **0.725** | -0.074 |
| Sediment pH | -0.412 | -0.310 | -0.032 | **0.841** | 0.167 |
| Sediment electrical conductivity | **0.771** | 0.234 | -0.126 | **-0.564** | 0.133 |
| Plant traits |  |  |  |  |  |
| Maximal rate of photosynthetic oxygen evolution | 0.199 | -0.085 | 0.203 | -0.164 | -0.047 |
| Total antioxidant capacity | 0.104 | 0.055 | 0.086 | -0.147 | 0.013 |
| Anthocyanin content | 0.028 | -0.533 | -0.009 | -0.072 | -0.05 |
| Carotenoid content | 0.418 | -0.215 | -0.034 | 0.039 | -0.033 |
| Chlorophyll *a* content | 0.073 | 0.083 | 0.046 | 0.032 | -0.04 |
| Chlorophyll *b* content | -0.346 | -0.035 | 0.017 | -0.074 | 0.048 |
| F_0_ at noon | 0.010 | 0.066 | 0.095 | 0.079 | 0.055 |
| F_0_ at sunrise | 0.120 | 0.019 | -0.077 | -0.056 | 0.022 |
| F_m_ at noon | -0.089 | 0.017 | 0.077 | 0.045 | 0.085 |
| F_m_ at sunrise | 0.099 | 0.006 | -0.100 | 0.011 | 0.081 |
| F_m_’ at noon | -0.132 | -0.200 | 0.096 | -0.17 | -0.002 |
| F_m_’ at sunrise | -0.086 | 0.118 | -0.102 | -0.035 | 0.085 |
| F_s_ at noon | -0.162 | -0.164 | 0.109 | 0.045 | -0.098 |
| F_s_ at sunrise | -0.098 | -0.330 | -0.104 | -0.051 | 0.015 |
| F_v_ at noon | -0.118 | 0.002 | 0.072 | 0.038 | 0.092 |
| F_v_ at sunrise | 0.094 | 0.004 | -0.039 | -0.035 | 0.028 |
| F_v_/F_m_ at noon | 0.008 | -0.033 | 0.001 | -0.057 | 0.042 |
| F_v_/F_m_ at sunrise | 0.044 | -0.011 | 0.154 | -0.314 | 0.042 |
| Apical leaf growth rate | 0.147 | 0.121 | 0.137 | -0.055 | -0.092 |
| MDA content | 0.194 | 0.166 | 0.107 | -0.174 | -0.124 |
| NPQ at noon | 0.144 | 0.434 | -0.363 | 0.454 | 0.395 |
| NPQ at sunrise | 0.404 | -0.448 | 0.110 | -0.095 | -0.025 |
| Polyphenol content | 0.029 | -0.105 | -0.028 | 0.394 | 0.068 |
| Φ_PSII_ at noon | 0.191 | -0.241 | 0.01 | -0.289 | 0.096 |
| Φ_PSII_ at sunrise | 0.024 | **0.465** | 0.203 | -0.164 | -0.047 |

**Table S5** Eigenvalues, proportion of variance explained and regression coefficients in a Canonical Correspondence Analysis (CCA) for canonical axes against standardized variables for ordination of plant trait responses relative to environmental conditions in winter and summer for *Spartina densiflora* *× maritima* in Guadiana salt marshes (Southwest Iberian Peninsula). Correlations with scores > ± 0.450 are marked in bold

| *Spartina densiflora × maritima* | Axis 1 | Axis 2 | Axis 3 | Axis 4 | Axis 5 |
| --- | --- | --- | --- | --- | --- |
| Eigenvalues | 0.026 | 0.018 | 0.008 | 0.002 | 0.000 |
| Variance explained | 0.361 | 0.247 | 0.108 | 0.025 | 0.006 |
| Environmental variables |  |  |  |  |  |
| Average daily air temperature | **0.504** | **0.472** | **0.677** | -0.242 | -0.083 |
| Sediment redox potential | -0.150 | 0.271 | 0.216 | **0.875** | 0.302 |
| Sediment water content | 0.390 | **0.740** | 0.329 | -0.439 | -0.020 |
| Sediment pH | **-0.670** | -0.217 | -0.438 | 0.166 | **-0.534** |
| Sediment electrical conductivity | **0.972** | 0.047 | 0.188 | 0.120 | -0.060 |
| Plant traits |  |  |  |  |  |
| Maximal rate of photosynthetic oxygen evolution | -0.044 | -0.113 | -0.331 | 0.065 | 0.052 |
| Total antioxidant capacity | -0.160 | -0.199 | -0.241 | 0.028 | -0.004 |
| Anthocyanin content | -0.403 | **-0.525** | -0.572 | 0.359 | -0.014 |
| Carotenoid content | **0.548** | -0.329 | -0.025 | 0.011 | -0.003 |
| Chlorophyll *a* content | 0.011 | 0.074 | -0.009 | -0.017 | 0.010 |
| Chlorophyll *b* content | 0.000 | 0.239 | -0.043 | 0.047 | -0.009 |
| F_0_ at noon | -0.030 | -0.082 | -0.145 | -0.060 | 0.011 |
| F_0_ at sunrise | -0.031 | -0.135 | -0.064 | -0.033 | 0.043 |
| F_m_ at noon | -0.090 | -0.083 | -0.081 | -0.048 | -0.012 |
| F_m_ at sunrise | 0.042 | -0.050 | 0.021 | 0.024 | 0.027 |
| F_m_’ at noon | -0.291 | -0.160 | 0.131 | 0.015 | -0.011 |
| F_m_’ at sunrise | 0.241 | 0.091 | 0.225 | -0.027 | -0.036 |
| F_s_ at noon | -0.298 | -0.148 | 0.149 | 0.024 | -0.001 |
| F_s_ at sunrise | 0.001 | -0.142 | -0.115 | 0.179 | -0.037 |
| F_v_ at noon | -0.104 | -0.084 | -0.065 | -0.045 | -0.017 |
| F_v_ at sunrise | 0.059 | -0.030 | 0.042 | 0.037 | 0.023 |
| F_v_/F_m_ at noon | -0.042 | -0.048 | -0.088 | -0.011 | -0.005 |
| F_v_/F_m_ at sunrise | -0.004 | -0.035 | -0.080 | 0.002 | -0.002 |
| Apical leaf growth rate | 0.070 | 0.088 | 0.052 | 0.014 | 0.021 |
| MDA content | 0.034 | 0.009 | -0.222 | -0.036 | -0.114 |
| NPQ at noon | 0.355 | 0.098 | **-0.847** | -0.16 | 0.054 |
| NPQ at sunrise | **-0.705** | -0.227 | -0.48 | -0.093 | 0.087 |
| Polyphenol content | -0.151 | -0.210 | -0.224 | 0.053 | 0.028 |
| Φ_PSII_ at noon | 0.108 | -0.153 | **-0.524** | -0.101 | -0.081 |
| Φ_PSII_ at sunrise | 0.282 | 0.130 | 0.287 | -0.106 | -0.021 |

**Table S6** Significant differences (General Linear Model, *P* < 0.05) between winter (W) and summer (S) for biochemical and ecophysiological and growth-related traits measured in individuals of *Spartina maritima*, *Spartina densiflora* and their reciprocal hybrids in the Guadiana Marshes (Southwest Iberian Peninsula)

|  | **Plant traits** | **Taxon** | | | |
| --- | --- | --- | --- | --- | --- |
|  |  | *S. maritima* | *S. maritima × densiflora* | *S. densiflora* | *S. densiflora × maritima* |
| Biochemistry | Anthocyanins (µg g DW^-1^) | W > S | W > S | W > S | W > S |
|  | Chl *a* (µg g DW^-1^) | S > W | - | - | - |
|  | Chl *b* (µg g DW^-1^) | - | - | - | - |
|  | Carotenoids (µg g DW^-1^) | W > S | - | - | - |
|  | MDA (nmol g DW^-1^) | - | S > W | - | - |
|  | Polyphenols (mg g DW^-1^) | - | W > S | - | W > S |
|  | Antioxidant capacity (mg g DW^-1^) | W > S | W > S | - | W > S |
| Ecophysiology and growth | Apical leaf growth (cm) | - | S > W | S > W | - |
|  | *V*_max_ (μmol O_2_ s^-1^ g^-1^ FW^-1^) | - | - | - | W > S |
|  | F_0_ at sunrise | - | - | - | - |
|  | F_v_ at sunrise | - | S > W | - | - |
|  | F_v_/F_m_ at sunrise | - | S > W | - | - |
|  | F_s_ at sunrise | - | - | W > S | - |
|  | F_m_’ at sunrise | S > W | S > W | - | S > W |
|  | ΦPSII at sunrise | S > W | S > W | S > W | S > W |
|  | NPQ at sunrise | - | W > S | - | W > S |
|  | F_0_ at noon | S > W | S > W | S > W | S > W |
|  | F_v_ at noon | - | S > W | - | - |
|  | F_v_/F_m_ at noon | - | S > W | - | - |
|  | F_s_ at noon | - | S > W | - | - |
|  | F_m_’ at noon | - | S > W | - | - |
|  | ΦPSII at noon | - | W > S | - | - |
|  | NPQ at noon | - | - | S > W | - |
